# Anomalous fractionation of mercury isotopes in the Late Archean atmosphere

**DOI:** 10.1038/s41467-020-15495-3

**Published:** 2020-04-06

**Authors:** Aubrey L. Zerkle, Runsheng Yin, Chaoyue Chen, Xiangdong Li, Gareth J. Izon, Stephen E. Grasby

**Affiliations:** 1 School of Earth and Environmental Sciences and Centre for Exoplanet Science, University of St Andrews, St Andrews, Fife, KY16 9AL Scotland UK; 2 State Key Laboratory of Ore Deposit Geochemistry, Institute of Geochemistry, Chinese Academy of Sciences, Guiyang, 550081 China; 3 State Key Laboratory of Environmental Geochemistry, Institute of Geochemistry, Chinese Academy of Sciences, Guiyang, 550081 China; 4 Department of Civil and Environmental Engineering, The Hong Kong Polytechnic University, Hung Hom, Kowloon Hong Kong; 5 Geological Survey of Canada, Calgary Natural Resources Canada, 3303 33rd Street NW, Calgary, AB T2L 2A7 Canada; 6 Present Address: Department of Earth, Atmospheric & Planetary Sciences, Massachusetts Institute of Technology, Cambridge, MA USA

**Keywords:** Element cycles, Geochemistry

## Abstract

Earth’s surface underwent a dramatic transition ~2.3 billion years ago when atmospheric oxygen first accumulated during the Great Oxidation Event, but the detailed composition of the reducing early atmosphere is not well known. Here we develop mercury (Hg) stable isotopes as a proxy for paleoatmospheric chemistry and use Hg isotope data from 2.5 billion-year-old sedimentary rocks to examine changes in the Late Archean atmosphere immediately prior to the Great Oxidation Event. These sediments preserve evidence of strong photochemical transformations of mercury in the absence of molecular oxygen. In addition, these geochemical records combined with previously published multi-proxy data support a vital role for methane in Earth’s early atmosphere.

## Abstract (web summary)

Earth’s surface underwent a dramatic transition ~2.3 billion years ago when atmospheric oxygen first accumulated during the Great Oxidation Event. Here, the authors find that biogenic methane and volcanic emissions played a vital role in the reduced Late Archean atmosphere.

## Introduction

Earth’s atmosphere has undergone significant changes during the 4.5 billion-year history of the planet, from fully anoxic and reducing in the Archean to the oxygen-rich atmosphere of today. Molecular oxygen is thought to have first become abundant in Earth’s atmosphere during the Great Oxidation Event (GOE), some ~2.3 billion years ago (Ga)^1,2^. However, recent evidence indicates that this change may not have been unidirectional^3,4^ and that dynamic atmospheric transitions could have occurred prior to the GOE^5,6^.

The utility of individual geochemical proxies for determining the composition of the atmosphere is limited, since most redox proxies reflect oceanic conditions^7^ or are only indirectly tied to atmospheric chemistry through an inferred connection with oxidative weathering^5,8^. Currently, the only geochemical proxy that can provide direct constraints on atmospheric chemistry under reducing conditions is that of the four stable isotopes of sulfur (^32^S, ^33^S, ^34^S, and ^36^S). The sulfur isotope record of sedimentary sulfides and sulfates prior to ~2.3 Ga is characterized by large magnitude Δ^33^S and Δ^36^S values which represent S isotope compositions that deviate significantly from those produced during the majority of chemical and biological processes^1^. The preservation of these S isotope signals, termed sulfur mass-independent fractionation (S-MIF), in Archean and earliest Proterozoic rocks is considered to be the strongest line of continuous evidence for low oxygen levels in the Earth’s early atmosphere (<10^−5^ times present atmospheric levels^9^). This is because low oxygen levels are a prerequisite for the production of S-MIF in the atmosphere, the transfer of the S-MIF to Earth’s surface, and the preservation of S-MIF in the geologic record. Despite the importance of S-MIF in providing upper limits on atmospheric oxygen levels, significant uncertainties remain about the specific reactions that generate this signal and how they respond in reducing atmospheres.

Mercury has seven stable isotopes (^196^Hg, ^198^Hg, ^199^Hg, ^200^Hg, ^201^Hg, ^202^Hg, and ^204^Hg) that are fractionated in the environment by both mass-dependent (Hg-MDF, denoted as δ^202^Hg) and mass-independent (Hg-MIF, denoted as Δ^199^Hg, Δ^200^Hg, and Δ^201^Hg) processes. Like sulfur, the atmosphere and oceans play critical roles in the global cycling of Hg. In the modern Hg cycle, gaseous Hg(0) represents >90% of the atmospheric Hg pool and has sufficiently long atmospheric residence time (0.5–2 years) for global transport^10^. Gaseous Hg(0) can be removed from the atmosphere through transformation to gaseous oxidized Hg(II) species, which are particle reactive and soluble, and thus more easily deposited through wet and dry deposition; or through direct uptake by vegetation and adsorption by soils, followed by oxidation to Hg(II)^11–13^. Mercury undergoes complex cycling between the atmosphere, land, and ocean, but its ultimate burial is typically to ocean sediments (e.g., as reviewed in ref. ^14^).

Mercury isotopes have been shown to be a useful tracer in constraining changes in the sources and cycling of Hg in Earth history, especially during mass extinction events^11,15–18^. Hg-MDF occurs during the majority of biogeochemical Hg cycling processes, but Hg-MIF in natural environments is primarily associated with photochemical processes in the atmosphere and surface waters^19–21^. MIF-bearing Hg deposited in the oceans complexes with organic matter and sulfur ligands, and can be preserved in sediments, where these signatures appear to be largely resistant to postdepositional alteration^11,15–17,22^. Hg-MIF preserved in sediments spanning Earth history can therefore provide insight into Hg cycling processes, particularly in the Precambrian in the absence of a significant terrestrial biosphere (e.g., ref. ^23^). Prior to the evolution of land plants, continents lacked significant terrestrial vegetation and soil development, such that volcanic Hg(0) would have been the sole source of Hg to the atmosphere^22^, and Hg cycling would have been largely controlled by abiotic transformations between the atmosphere and oceans. The lack of oxygen in the Archean atmosphere and ocean would have resulted in significantly different atmospheric reactions and burial of Hg in sediments as well. However, to date no detailed study of Hg isotopes has been conducted to examine the cycling of Hg and manifestation of Hg-MIF signals in Archean sediments. Here we use Hg-MIF data from Late Archean sediments to provide important constraints on Hg cycling and atmospheric chemistry in the immediate run-up to the GOE. These sediments preserve evidence of strong atmospheric transformations of Hg in an anoxic atmosphere. Combined with previous records of S-MIF, changes in Hg-MIF also support an important role for methane and hydrocarbon haze formation in the Late Archean atmosphere.

## Results and discussion

### Hg isotopes in Late Archean sediments

**Fig. 1:**
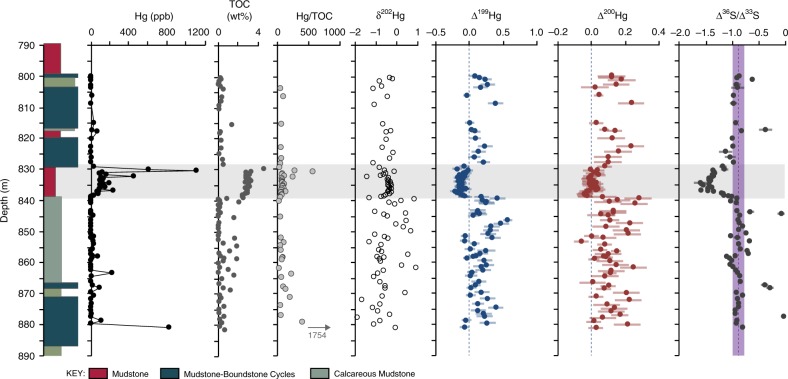
Lithologic and geochemical data for the studied section. Data are listed in Supplementary Data 1, and include Hg concentrations (ppb), total organic carbon (TOC, wt%), Hg/TOC ratios for samples with TOC > 0.2 wt% (ppb/wt%), Hg isotope values (δ^202^Hg, Δ^199^Hg, and Δ^200^Hg, in ‰), and pyrite S-MIF data (Δ^36^S/Δ^33^S; from ref. ^26^). Uncertainties on Hg isotope data correspond to the larger value of either the measurement uncertainty of replicate digests of MESS-2 or the uncertainty of repeated measurements of UM-Almadén. Uncertainty on Δ^36^S/Δ^33^S was computed from the larger of the internal or external uncertainties associated with the raw Δ^33^S and Δ^36^S data (as previously reported^26^). Analytical uncertainties for additional data are encompassed within each individual data point. The gray shaded area indicates the interval where changes in Δ^36^S/Δ^33^S correspond to changes in Hg-MIF values; the vertical purple line illustrates the background Archean Δ^36^S/Δ^33^S ratio; vertical dashed lines also illustrate 0‰ in Δ^199^Hg and Δ^200^Hg.

We studied sediments from the lower part of the Upper Nauga Formation, sampled from core GKF01 drilled by the Agouron Institute in the Griqualand West Basin, South Africa^24^ (SI, Supplementary Fig. 1). The core consists mainly of mudstones and dolostones deposited in a deep water slope environment of the Cambellrand-Malmani carbonate platform, as described in detail by Schröder et al.^24^ (Supplementary Fig. 2). Precise U-Pb zircon ages from associated volcani-clastic sediments place the base of the core at ~2.65 ± 0.08 Gyr ago and the top of the core (within the Upper Nauga) at ~2.5 Gyr ago^25^. Our interval of study spans several mudstone-dominated lithologies of the Upper Nauga Fm (Fig. 1). These and other mudstones within the Upper Nauga Fm have been interpreted to represent deep subtidal sediments deposited below storm weather wave base, and generally associated with basinal deepening and/or higher input of fine-grained detritus^24^. The GKF01 core has previously been the focus of extensive geological and geochemical characterization, including high-resolution Fe speciation and S-MIF analyses (Supplementary Fig. 2)^6,24,26^.

**Fig. 2:**
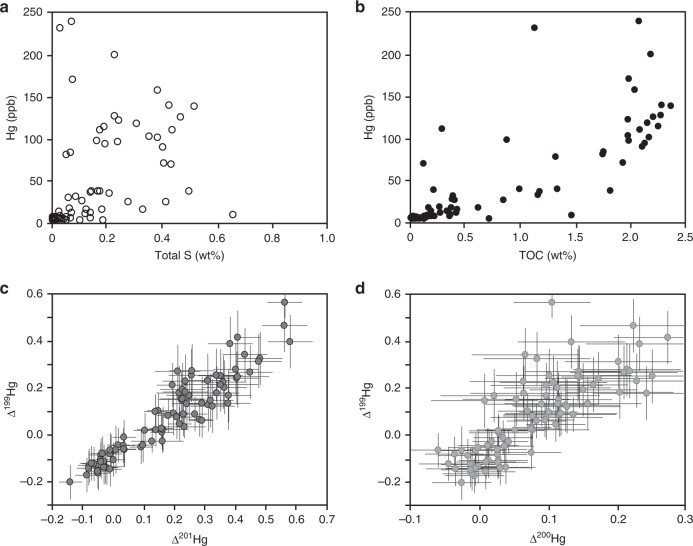
Cross plots of geochemical data. Uncertainties on Hg isotope data correspond to the larger value of either the measurement uncertainty of replicate digests of MESS-2 or the uncertainty of repeated measurements of UM-Almadén; analytical uncertainties for additional data are encompassed within each individual data point. These plots demonstrate a lack of correlation between Hg (ppb) and total sulfur content (S wt%) (**a**), and generally positive correlations between Hg (ppb) and total organic carbon (TOC, wt%) (**b**), Δ^199^Hg and Δ^201^Hg (both in ‰) (**c**), and Δ^199^Hg and Δ^200^Hg (both in ‰) (**d**).

Across an interval from ~840 to 830 m in the core, mercury concentrations rise from ≤10 ppb to an average of 183 ppb, in association with an increase in total organic carbon (TOC) (Fig. 1; Supplementary Data 1). High levels of sedimentary Hg above the average shale values (>62 ppb^22^) could be due to either increased input of Hg to the basin or to increased burial efficiency of Hg into the sediments. Hg in the modern oceans has a high affinity for both organic matter and organic sulfur ligands (thiols). The Hg content in these rocks shows no correlation with total sulfur content (Fig. 2a), and previously published paleoredox data show no evidence for euxinic depositional conditions in this interval (Supplementary Fig. 2; ref. ^6,26^), discounting enhanced scavenging by sulfur ligands as a cause for increased Hg burial. Instead, the Hg content in these rocks is positively correlated with wt% TOC (Fig. 2b), although the Hg/TOC values show no systematic trends through the study interval (Fig. 1). Since volcanic emissions commonly (though not always) shift Hg/TOC ratios in sediments^22^, these data might indicate that changes in Hg concentrations throughout this section are dominantly controlled by variations in TOC rather than volcanic input. In contrast, three samples exhibit very high Hg concentrations (with Hg > 1100 ppb) and correspondingly large spikes in Hg/TOC ratios (Fig. 1), consistent with a major influx of Hg from an external source, such as increased volcanic activity^22^. We explore the implications of a volcanic Hg source on Hg isotope trends in the discussion below.

Hg-MDF provides limited constraints on Hg cycling throughout this time period. We measure δ^202^Hg values from −1.9 to +0.9‰ (Fig. 1) in this section, spanning the range of δ^202^Hg values previously reported for marine sediments (−2.5 to 1‰; ref. ^20,22^). However, δ^202^Hg alone is generally insufficient to identify specific sources and Hg cycling reactions, because Hg-MDF is ubiquitous and occurs during all biological reactions (e.g., reduction, methylation, and demethylation), abiotic reactions (e.g., chemical reduction, photoreduction, and oxidation), and physical processes (e.g., volatilization, evaporation, adsorption, and dissolution) (as reviewed by ref. ^20,27,28^). Equilibrium partitioning and speciation can also cause significant Hg-MDF in natural samples (as reviewed by ref. ^20,27,28^), further complicating interpretations of sedimentary δ^202^Hg values. Since we lack a complete understanding of all the Hg-MDF processes in the Late Archean ocean and atmosphere, it is therefore difficult to interpret the variations of δ^202^Hg further in this study (as in previous studies^15,23^).

In contrast, Hg-MIF provides more clear constraints on Hg cycling in the Archean, as it mainly occurs during photochemical processes with little contribution from complex biogeochemical cycling. Notably, significant changes in Hg-MIF occur throughout this section, for both odd- and even-number Hg isotopes (Fig. 1). We observe significant odd-number Hg-MIF, with Δ^199^Hg values from −0.2 to 0.6‰ (Fig. 1) and a Δ^199^Hg/Δ^201^Hg ratio of ≈1 (Fig. 2c). MIF of odd-number Hg isotopes, with Δ^199^Hg/Δ^201^Hg ratios ≈1, has previously been observed in various geochemical pools near Earth’s surface (see^20^ and references therein), in samples mainly consisting of inorganic Hg species (Supplementary Fig. 3), and this signature has been attributed to the photoreduction of aqueous Hg(II) to Hg(0)^19^. Aqueous Hg(II) photoreduction produces negative Δ^199^Hg and Δ^201^Hg values in the product Hg(0), and positive Δ^199^Hg and Δ^201^Hg values in the residual Hg(II). Modeling studies suggest that Hg(II) photoreduction occurring in surface waters and aerosols plays an important role in generating odd-number Hg-MIF in natural samples^29,30^. Rain and seawater samples^31–35^, which mainly contain Hg(II) species, are characterized by positive odd-number Hg-MIF, whereas terrestrial reservoirs (e.g., plants and soils) that primarily accumulate Hg(0) are characterized by negative odd-number Hg-MIF (as summarized by^20^) (Supplementary Fig. 3). Odd-number Hg-MIF has also been observed in ocean sediments deposited since the Mesoproterozoic^16,17,23,36–38^. Specifically, coastal sediments tend to have negative odd-number Hg-MIF due to a major contribution from terrestrial Hg sources, while offshore sediments mainly show positive odd-number Hg-MIF due to a major contribution from atmospheric Hg(II) deposition^37^. In the sediments we examined, the upper and lower intervals of the section show positive Δ^199^Hg values, whereas organic-rich mudstones within the interval from ~830 to 840 m depth are characterized by slightly negative Hg-MIF or zero Δ^199^Hg values from −0.2 to −0.01‰ (with 2σ of 0.07‰) (Fig. 1).

Significant changes in even-number Hg isotopes, with Δ^200^Hg values from −0.1 to +0.3‰ (Fig. 1), are also preserved throughout this section. Unlike odd-number Hg-MIF, large MIF of even-number Hg isotopes has only been observed in modern atmospheric samples. For instance, rain samples, which mainly contain oxidized Hg(II) species, are characterized by positive Δ^200^Hg signals, whereas gaseous Hg(0) shows negative Δ^200^Hg signals^32–35^ (Supplementary Fig. 3). For this reason, seawater and ocean sediments sometimes have slightly positive Δ^200^Hg due to wet deposition of atmospheric Hg(II)^17,31,37^. Photoreduction of aqueous Hg(II) does not induce even-numbered Hg-MIF, so these anomalies are thought to be produced exclusively during photooxidation of gaseous Hg(0) in the atmosphere^21,32,34^. The majority of our studied section shows positive Δ^200^Hg values, although the organic-rich mudstones within the interval from ~830 to 840 m depth are characterized by near zero Δ^200^Hg values, from −0.04 to +0.08‰ (with 2σ of 0.06‰) (Fig. 1).

### Late Archean Hg isotope mass-independent fractionation

**Fig. 3:**
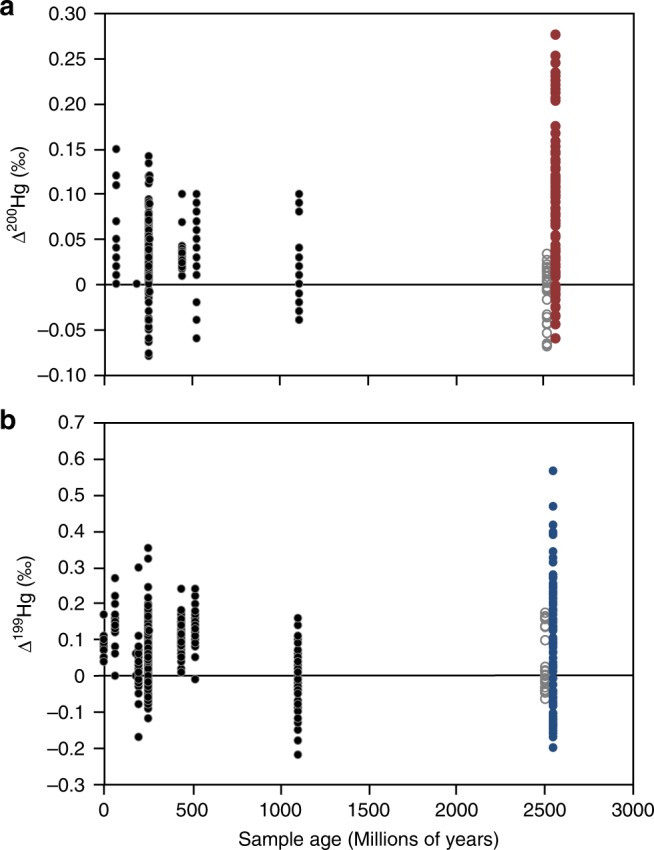
Temporal plot of mercury isotope data from the rock record. These include Δ^200^Hg (in ‰, **a**) and Δ^199^Hg (in ‰, **b**). Our data are shown as red dots (for Δ^200^Hg) and blue dots (for Δ^199^Hg). Previously published Proterozoic and Phanerozoic data (shown as black dots) are compiled from^15–18,20,23,36,38,41,55–57^. The only previously published Hg-MIF data from Archean sediments (shown as gray open circles) were from the Mt McRae shale^20^, which have been controversially associated with transient atmospheric oxygen before the GOE^5^.

The positive Hg-MIF measured throughout the majority of this section record some of the highest Δ^199^Hg values (up to 0.6‰) and Δ^200^Hg values (up to 0.3‰) reported in ocean sediments to date^16,17,22,36–38^ (Fig. 3). Since Δ^200^Hg anomalies are exclusively produced in the atmosphere, the strong correlation between Δ^199^Hg and Δ^200^Hg observed in these samples (Fig. 2d) also implies that both signals were mainly formed in the atmosphere. The large magnitude of these Hg-MIF values suggests that Hg(II) photoreduction and Hg(0) photooxidation were more intense when these sediments were deposited, likely due to the lack of significant levels of molecular oxygen in the Late Archean atmosphere. This follows because molecular oxygen is required for ozone formation, and ozone blocks ultraviolet radiation that can drive photochemical reactions near the Earth’s surface.

While atmospheric deposition of Hg(0) carrying negative Hg-MIF signals could also have occurred during this time, the generally positive Δ^199^Hg and Δ^200^Hg values in our samples suggest that atmospheric Hg(II) deposition was the dominant input of Hg to these sediments. Once deposited in the oceans, Hg(II) carrying positive Hg-MIF signals would subsequently have been scavenged by organic particles and transported to the seafloor. Hg(II) photoreduction could also have occurred in surface seawater, contributing to the shift in the Δ^199^Hg values; however, the magnitude of this shift is generally limited in the present-day oceans, because photoreduction of Hg in oceans is suppressed by excessive chloride ligands which form strong complexes with Hg(II)^39^. A recent study in the modern ocean demonstrated no significant difference in Δ^199^Hg in seawater at different depths, suggesting that Hg(II) photoreduction does not significantly alter water column Δ^199^Hg values, at least in the modern oceans^31^.

**Fig. 4:**
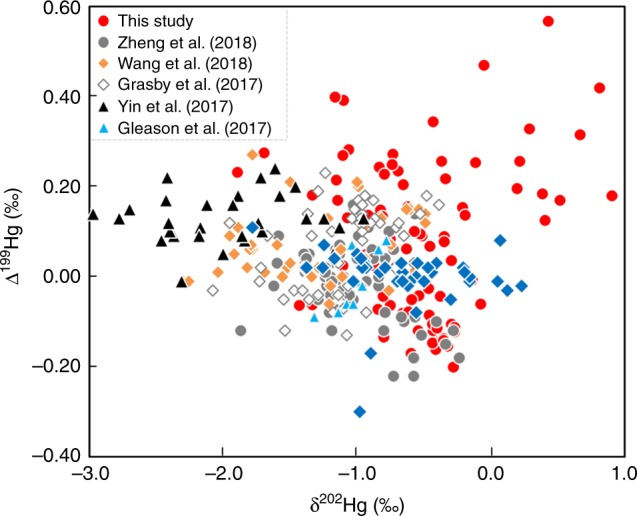
Cross plots of mercury isotope data from marine sediments. These include δ^202^Hg versus ∆^199^Hg from late Late Archean sediments (our data) compared with Late Mesoproterozoic shales from Africa^23^, Early Cambrian shales from China^17^, end-Permian sediments from China^16,41^, end-Triassic shelf sediments from North America^58^, and Eocene shales from the Arctic Basin^40^.

During laboratory experiments, photochemical oxidation or reduction of Hg generally produces trends in Hg-MDF that are correlated with Hg-MIF. For example, a correlation between δ^202^Hg and Δ^199^Hg, with Δ^199^Hg/δ^202^Hg of ~1.2, is generally associated with photochemical reduction reactions induced in the laboratory^15–17,23,40,41^. The δ^202^Hg values in our study are not correlated with either Δ^199^Hg or Δ^200^Hg; however, we would not expect any correlation to be preserved in natural samples, given that (as above) numerous physical, chemical, and biological processes occurring in the water column and sediments can produce MDF that would shift the sedimentary δ^202^Hg without affecting the Δ^199^Hg or Δ^200^Hg. This assertion is supported by previous studies of ocean sediments, which have also shown no correlation between δ^202^Hg and Δ^199^Hg^15–17,23,40,41^ (Fig. 4). This comparison with previously published data also demonstrates that the majority of our data appear to occupy a different field in δ^202^Hg versus Δ^199^Hg space than marine sediments from more recent time periods in Earth history, pointing to some fundamental difference(s) in Hg-MIF generation in anoxic versus oxygenated atmospheres. A detailed examination of the potential mechanisms for this difference is beyond the scope of the current study, but should provide a critical target for future work.

Interestingly, enhanced Hg concentrations within the organic-rich mudstone interval from ~830 to 840 m depth in core GKF01 are associated with significant changes in both Δ^199^Hg and Δ^200^Hg, to zero or slightly negative Hg-MIF values. As mentioned above, negative Δ^199^Hg and Δ^200^Hg values are generally characteristic of gaseous Hg(0). Runoff of vegetation and terrestrial particles, which adsorb gaseous Hg(0), are the main contributors of negative Hg-MIF to modern sediments; however, these sources would have been negligible in the Archean due to the absence of vegetation and significant terrestrial soils^42^. Consequently, we suggest that the following scenarios could be responsible for shifting Δ^199^Hg and Δ^200^Hg values over this interval: enhanced sequestration of atmospheric Hg(0) to the sediments by thiols and sulfides that were enriched in the surface ocean as a result of photic zone euxinia (e.g., as proposed for the Mesoproterozoic^23^) (scenario 1); enhanced terrestrial input of geogenic Hg with no MIF signals (scenario 2); enhanced volcanic input of Hg with no MIF signals (scenario 3); or changes in production or deposition of Hg-MIF associated with changes in atmospheric chemistry (scenario 4).

As noted above, the lack of evidence for water column euxinia^26,43^ (Supplementary Fig. 2) along with a lack of correlation between Hg-MIF and total sulfur content (Fig. 2a) suggests that enhanced sequestration by thiols is unlikely, allowing the elimination of scenario 1. Enhanced terrestrial influx of Hg, as invoked in scenario 2, is inconsistent with lithological and elemental evidence. Namely, the facies change towards more organic carbon-rich mudstones suggests a move toward a more distal depositional environment^24^, which is inconsistent with additional terrestrial runoff from land. Elemental proxy data also support basinal deepening and a general decrease in terrestrial detritus (Supplementary Fig. 4). Given that changes in Si/Al are generally interpreted to imply a change in terrestrial provenance (excluding biological Si-cycling), if we take Si/Al as a broad indicator, the decrease in Ti and Zr at the expense of Al (as shown in Supplementary Fig. 4) is consistent with deeper water deposition and entrainment of heavy minerals (rutile and zircon) in nearshore settings. The increase in K/Al further suggests the increased significance of clays in deeper water settings. We also note that limited data from the similar organic-rich mudstone unit up-core do not feature similar Hg isotope systematics, further arguing against a simple facies control on this signature.

An increase in volcanic Hg input across this interval (scenario 3) cannot explain the change in Hg-MIF values either. Hg concentrations and Hg/TOC remain generally low in these strata, suggesting that even if there were volcanism it did not greatly perturb the Hg cycle. Discrete peaks in Hg levels and Hg/TOC could indicate periodically enhanced volcanic Hg emissions^22^, but REE patterns from this interval further suggest that volcanism was not pervasive^24^. Regardless, the lack of correlation between the peak in Hg/TOC and the change in Hg-MIF means that even if the Hg/TOC peak was volcanically derived, the volcanism was not solely responsible for the Hg cycle perturbation.

Given the lack of evidence for enhanced euxinia or terrestrial input, and only discrete evidence for enhanced volcanism, we suggest that the changes in Hg-MIF within this interval were largely due to a change in atmospheric chemistry (scenario 4). In particular, the slightly negative Δ^199^Hg values at the bottom of this interval (Fig. 1) support the increased importance of atmospheric Hg(0) as a source of Hg to the oceans. Since only Hg(II) is particle reactive and rains out into the oceans, this change would have required the atmospheric Hg(0) pool to be oxidized and deposited from the atmosphere without further changes to Hg isotope values. The presence of large magnitude S-MIF throughout this section (Supplementary Fig. 2) precludes the involvement of oxygen or ozone as a major component of the atmosphere^9^. In addition, Hg(0) is mainly oxidized by halogens (iodine and bromine), even in the modern, oxygenated atmosphere^44^. This trend would therefore imply that some additional change in reducing atmospheric chemistry occurred.

Previously published multi-proxy geochemical data from this section provide further insight into atmospheric chemistry during this time interval. Similar to Δ^199^Hg and Δ^200^Hg, S-MIF values in sedimentary pyrite and δ^13^C values in organic carbon show large changes across the same part of this section (Fig. 1). In particular, Δ^36^S/Δ^33^S ratios change from a background of approximately −0.9 to much steeper slopes of approximately −1.5^26^ in correlation with a negative excursion in δ^13^C_org_ (Fig. 1; Supplementary Fig. 2). Similar changes in Δ^36^S/Δ^33^S and δ^13^C_org_ have been documented in multiple horizons within the GKF01 core, all independent of facies changes (Supplementary Fig. 2; see also Fig. S3 from ref. ^26^). These trends have now been correlated between five cores from South Africa and Western Australia^45^, and are shown to be conserved at the grain scale (Supplementary Fig. 2; ref. ^46^). The consistency of these changes in S-MIF signals across multiple spatial scales has been used to argue for a global atmospheric driver^6,26,47^. The largest Δ^200^Hg values we measure are correlated with the most negative Δ^36^S/Δ^33^S ratios in this section (Supplementary Fig. 5), suggesting a common mechanism.

Coupled changes in S-MIF and δ^13^C_org_ in the Late Archean have previously been interpreted to reflect changes in atmospheric chemistry and transparency induced by formation of a hydrocarbon-rich haze at high atmospheric CH_4_:CO_2_^6,26,45^. Photochemical models of S-MIF formation in the Archean atmosphere have further demonstrated the ability of organic haze to change atmospheric S-MIF signatures^6,26,47^. The changes in Hg-MIF across this interval could also be explained by organic haze formation, since organic aerosols could have induced deposition of gaseous Hg(0) via oxidation and sorption. Elemental Hg(0) can be oxidized and scavenged by low-molecular-weight organic compounds in the atmosphere, as demonstrated by consistent Hg/TOC ratios measured in modern precipitation^48^. Thus oxidation and sequestration of gaseous Hg(0) by organic particles in the haze could have transported these negative Hg-MIF signals to the oceans and sediments, consistent with the trends in Hg and TOC described above.

The continued presence of organic haze could have limited further Hg-MIF formation throughout the rest of this interval by limiting or changing the penetration of ultraviolet radiation and inhibiting the photochemical reactions of Hg, as the negative Hg-MIF-bearing Hg(0) was progressively stripped from the atmosphere. Previous studies have proposed that even-number Hg-MIF mainly forms in the tropopause. After photooxidation, Hg(II) carrying the positive Δ^200^Hg anomaly could be transported downward by stratosphere-to-troposphere incursion to the surface. In fact, even-number Hg-MIF of rain samples worldwide displays a general increase with latitude, confirming the upper atmosphere as the possible origin^49^. Photochemical models of the Archean atmosphere suggest that hydrocarbon haze would have formed very high in the stratosphere^50,51^, and therefore provides a viable alternative mechanism to shield even-number Hg-MIF formation in the tropopause, as well as odd-number Hg-MIF formation in the surface oceans and atmosphere^32,34^.

**Fig. 5:**
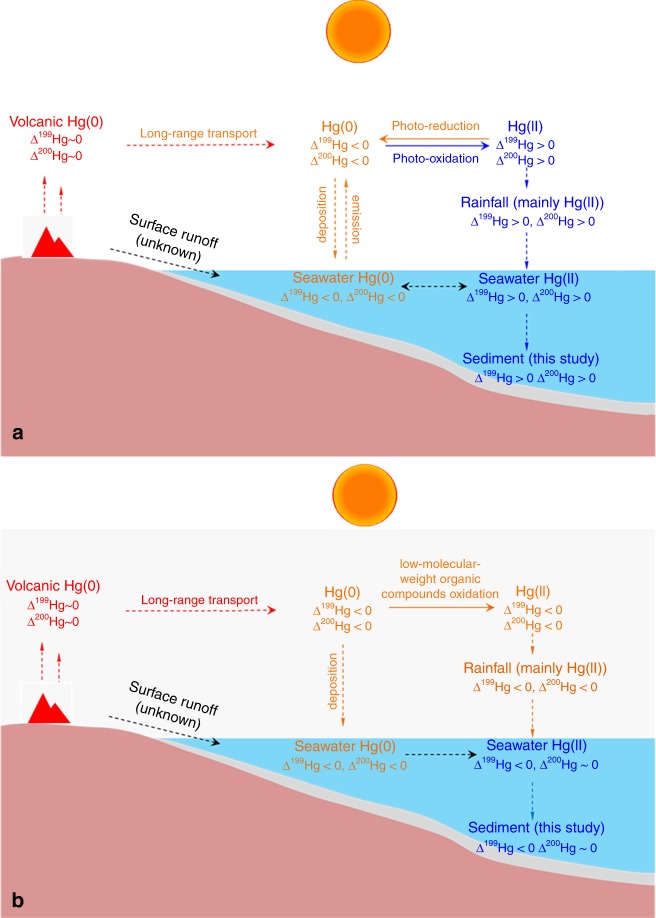
Proposed scenarios for observed mercury isotope fractionation trends. **a** Intense photochemical reactions in the absence of an ozone layer lead to the production of large Hg-MIF in the Archean atmosphere. The red color represents volcanic Hg(0), which has been the primary source of Hg to the Earth surface over geologic time. This volcanic Hg(0) is characterized by Δ^199^Hg ~0‰ and Δ^200^Hg ~0‰, and can undergo long-range transport in the atmosphere. Atmospheric Hg(0) was oxidized to gaseous Hg(II), and a fraction of that Hg(II) was photoreduced back to Hg(0). These photochemical processes produced gaseous Hg(0) with negative Δ^199^Hg and Δ^200^Hg values, and gaseous Hg(II) with positive Δ^199^Hg and Δ^200^Hg values. The orange color represents the deposition of gaseous Hg(0) to ocean surface and the reemission to the atmosphere. The light blue color represents the deposition of gaseous Hg(II) through precipitation, the scavenging of seawater Hg (mainly precipitated Hg(II)) by organic particles, and the burial of organic-bound Hg with mainly positive Δ^199^Hg and Δ^200^Hg signals in marine sediments. **b** In the presence of a hydrocarbon haze, nearly all Hg(0) was oxidized by low molecular weight organic compounds before deposition, scavenging, and burial. The red color represents volcanic Hg(0), which was mixed with background atmospheric Hg(0) carrying negative Δ^199^Hg and Δ^200^Hg values. The orange color represents nearly complete oxidation of Hg(0) to Hg(II), and therefore the deposited Hg(II) is characterized by negative Δ^199^Hg and Δ^200^Hg values. The light blue color represents the mixing of the deposited atmospheric Hg(II) with seawater Hg that is characterized with positive Δ^199^Hg and Δ^200^Hg values. The scavenging and the burial of these two forms of Hg produced marine sediments with slightly negative Δ^199^Hg and near zero Δ^200^Hg signals.

The scenario we propose for producing the observed trends in Hg-MIF is summarized in Fig. 5. During the Late Archean (Fig. 5a), intense photoreduction and photooxidation in the absence of ozone produced particle-reactive Hg(II) with large positive Δ^199^Hg and Δ^200^Hg that were deposited into the oceans and subsequently the sediments. These processes left the atmospheric Hg(0) pool with negative Δ^199^Hg and Δ^200^Hg, similar to the modern environment. During the hazy period (Fig. 5b), the negative Hg-MIF-bearing atmospheric Hg(0) was oxidized to Hg(II) by low-molecular-weight organic compounds and deposited to the ocean, which then diluted the positive MIF signals in seawater, and caused negative shifts of Δ^199^Hg and Δ^200^Hg in seawater and sediments. Decreased atmospheric transparency could have hindered further Hg-MIF production, until the haze cleared and background Hg-MIF production resumed.

These striking observations provide definitive support for Hg-MIF as a powerful paleoatmospheric proxy in marine sediments. We have shown that these data respond differently than S-MIF to changes in the chemistry of reducing atmospheres, and can thus provide independent, complementary constraints on the composition and evolution of atmospheric chemistry in the Archean. Combined, these records point to a dynamic Late Archean atmosphere on the run-up to the GOE, with at least two distinct atmospheric states responding to both volcanic emissions and methane production. Notably, these data support earlier suggestions of a vital role for the anaerobic biosphere and biogeochemical methane cycling in the evolution of Earth’s early atmosphere.

## Methods

### Elemental analyses

Samples from core GKF01, through the ~2.6–2.5 Ga Griqualand West Basin, South Africa^24^, were collected from the National Core Library at Donkerhoek (Pretoria, South Africa) (Supplementary Fig. 1), as described by Izon et al.^26^ Major elemental abundance data (listed in Supplementary Data 2) were determined by X-ray florescence analysis at the University of St Andrews, Scotland, following the protocol outlined by^52^. Here, aliquots of homogenized samples were pre-combusted at 1000 °C in ceramic crucibles to remove their volatile constituents, thus allowing loss on ignition to be calculated. The volatile-free sample residues were fused using a mixed lithium tetraborate (Li_2_B_4_O_7_; 20%) and lithium metaborate (LiBO_2_; 80 %) flux in Pt–Au crucibles. Ammonium iodide (NH_4_I) was used as a releasing agent. These fused samples were analyzed on a Spectro Xepos HE, with a 50 Watt end-window X-ray tube to excite the samples, and a 30 mm^2^ Si-drift detector with Peltier cooling, providing spectral resolution (FWHM) ≤ 155 eV at Mn K-alpha. Replicate analysis of a suite of standards (GSR1–152 6, SBC-1, OU-8, OU-6, and SD0-1) produced values that were inseparable from their certified values, equating to relative uncertainties of <3%.

Trace element analyses (listed in Supplementary Data 3) were also performed at the University of St Andrews. Here, 0.25 g aliquots of pre-combusted residues were mixed and fused with 1.25 g of an equal mixture of Li_2_B_4_O_7_ and LiBO_2_ in acid-cleaned Pt–Au crucibles. The resultant glass bead, after dissolution in 5% (vol/vol) ultra-pure nitric acid, was manipulated and prepared for analysis using a Thermo X series2 ICP-MS. Standardization was achieved via matrix-matched synthetic standards, while drift was corrected by internal normalization after Re, Ge, and Rh doping. Alongside unknowns, the same suite of major element standards was processed in an identical manner to the unknowns, producing data typically better than 10% of their certified values.

### Mercury isotope analyses

Total Hg concentrations (summarized in Supplementary Data 1) were determined by the RA-915+ Hg analyzer coupled with the PYRO-915+ attachment (Lumex, Russia), at Institute of Geochemistry, Chinese Academy of Sciences (IGCAS). Recoveries for standard reference materials GSS-5 (*n* = 3) and MESS-2 (*n* = 3) were between 94 and 112%, and coefficients of variation (triplicate analyses) were <8%.

At IGCAS, a double-stage tube furnace coupled with 40% anti aqua regia (HNO_3_/HCl = 2/1, v/v) trapping solutions was used for Hg preconcentration, prior to isotope analysis. This combustion system was developed and validated in a previous study^53^. For samples with high Hg concentrations (>25 ng g^−1^), an acid digestion method using aqua regia (HNO_3_/HCl = 1/3, v/v) was used in parallel to the combustion method^54^. MESS-2 was included in both sample preparation methods. All the solutions were diluted to ~0.5 ng mL^−1^ Hg in 10–20% (v/v) acids using 18.2 MΩ cm water, and analyzed by Neptune Plus multiple collector inductively coupled plasma mass spectrometer (Thermo Electron Corp, Bremen, Germany) at the University Research Facility in Chemical and Environmental Analysis (UCEA) in the Hong Kong Polytechnic University, following our recently published method^54^. The instrument was equipped with the HGX-200 system and an Aridus II Desolvating Nebulizer System (CETAC Technologies, USA) for Hg and Tl introduction, respectively. NIST SRM 997 Tl standard (50 ng mL^−1^) was used as an internal standard for simultaneous instrumental mass bias correction of Hg. The instrument was tuned using the NIST SRM 3133 Hg standard solution for maximum intensity of ^202^Hg signal using Ar gas flows, torch position, and lenses. The sensitivity for ^202^Hg was 1.8 V per ng mL^−1^ Hg. Total Hg in sample solutions was monitored by MC-ICP-MS using ^202^Hg signals. The signals for ^202^Hg were <0.01 V for acid blanks of both sample preparation methods, corresponding to <1% of that in sample solutions. The Hg recoveries of MESS-2 were 96–110% for the combustion method (*n* = 9), similar to that yielded by the acid digestion method (94–111%, *n* = 3). Total Hg values of the samples estimated by ^202^Hg signals were 92–119% to that measured by RA-915+ Hg analyzer. The larger variability of samples compared with MESS-2 (Supplementary Table 1) is potentially due to heterogeneities in Hg concentration of sample powders.

NIST SRM 3133 Hg standard solutions and UM-Almadén secondary standard solutions were prepared, with matrix and concentration matched within 10% to sample dilutions. Standard sample bracketing was performed with NIST SRM 3133 Hg standard solutions. Mercury isotope were reported following convention^19^, such as mass-dependent fractionation was expressed in δ^202^Hg notation in units of permil (‰) referenced to the NIST SRM 3133 Hg standard (analyzed before and after each sample):

1 $${\updelta}^{202}{\mathrm{Hg}}\left( ‰\right) = \left[ {\left( {{\,}^{202}{\mathrm{Hg/}}^{198}{\mathrm{Hg}}_{{\mathrm{sample}}}} \right)/\Big( {{\,}^{202}{\mathrm{Hg/}}^{198}{\mathrm{Hg}}_{{\mathrm{standard}}}} \Big) - 1} \right] \times 1000$$

Mass-independent fractionation is reported in Δ notation (Δ^xxx^Hg), which describes difference between the measured δ^xxx^Hg and the theoretically predicted δ^xxx^Hg value, in units of permil (‰), using the following formula:

2 $$\Delta ^{{\mathrm{xxx}}}{\mathrm{Hg}} \approx {\updelta}^{{\mathrm{xxx}}}{\mathrm{Hg}} - {\updelta}^{202}{\mathrm{Hg}}\, \times {\upbeta},$$

*β* is equal to 0.2520 for ^199^Hg, 0.5024 for ^200^Hg, and 0.7520 for ^201^Hg^19^.

Analytical uncertainty was estimated based on the replication of the UM-Almadén secondary standard solutions and full procedural analyses of MESS-2. As shown in Supplementary Table 1, results of UM-Almadén are consistent with recommended values^19^, and MESS-2 processed with the acid digestion and the oven combustion procedure showed identical isotope compositions which are consistent with recommended values^16,17^, thereby validating the methods. Uncertainties reported in this study correspond to the larger value of either the measurement uncertainty of replicate digests of MESS-2 or the uncertainty of repeated measurements of UM-Almadén.

## Supplementary information

Supplementary Information

Peer Review File

Description of Additional Supplementary Files

Supplementary Data 1

Supplementary Data 2

Supplementary Data 3

## Data Availability

All new data from this study are available in the Supplementary Data files; previously published data are available from the referenced works.
