# Supplementary material for: Anomalous fractionation of mercury isotopes in the Late Archean atmosphere
Source: Nat Commun. 2020 Apr 6;11:1709. doi: 10.1038/s41467-020-15495-3 (PMC7136252; doi:10.1038/s41467-020-15495-3)
Supplement: Supplementary file 1 — Supplementary Information [file 41467_2020_15495_MOESM1_ESM.pdf]

Supplementary Information for:

Anomalous fractionation of mercury isotopes in the Late Archean atmosphere

Aubrey L. Zerkle et al.

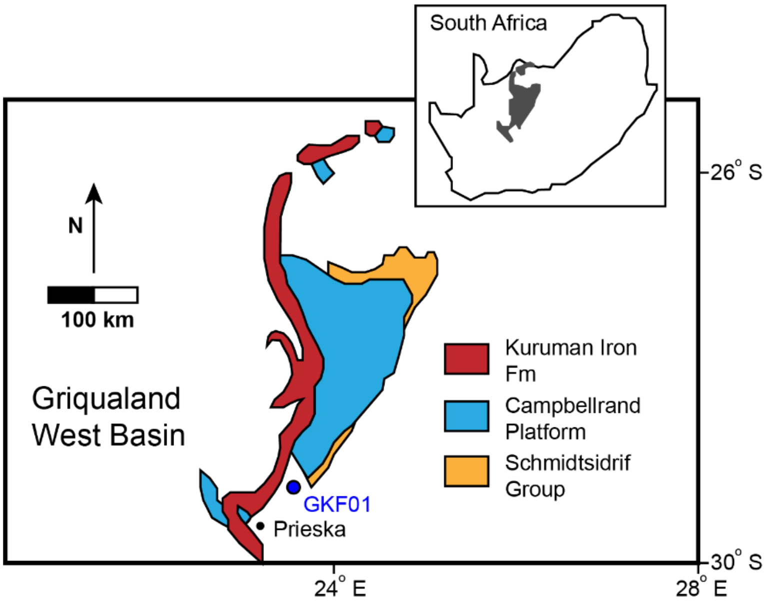

Supplementary Figure 1. Map of the Griqualand West Basin in South Africa. The position of the studied core GKF01 is shown in blue (modified from Schroder et al. [1]).

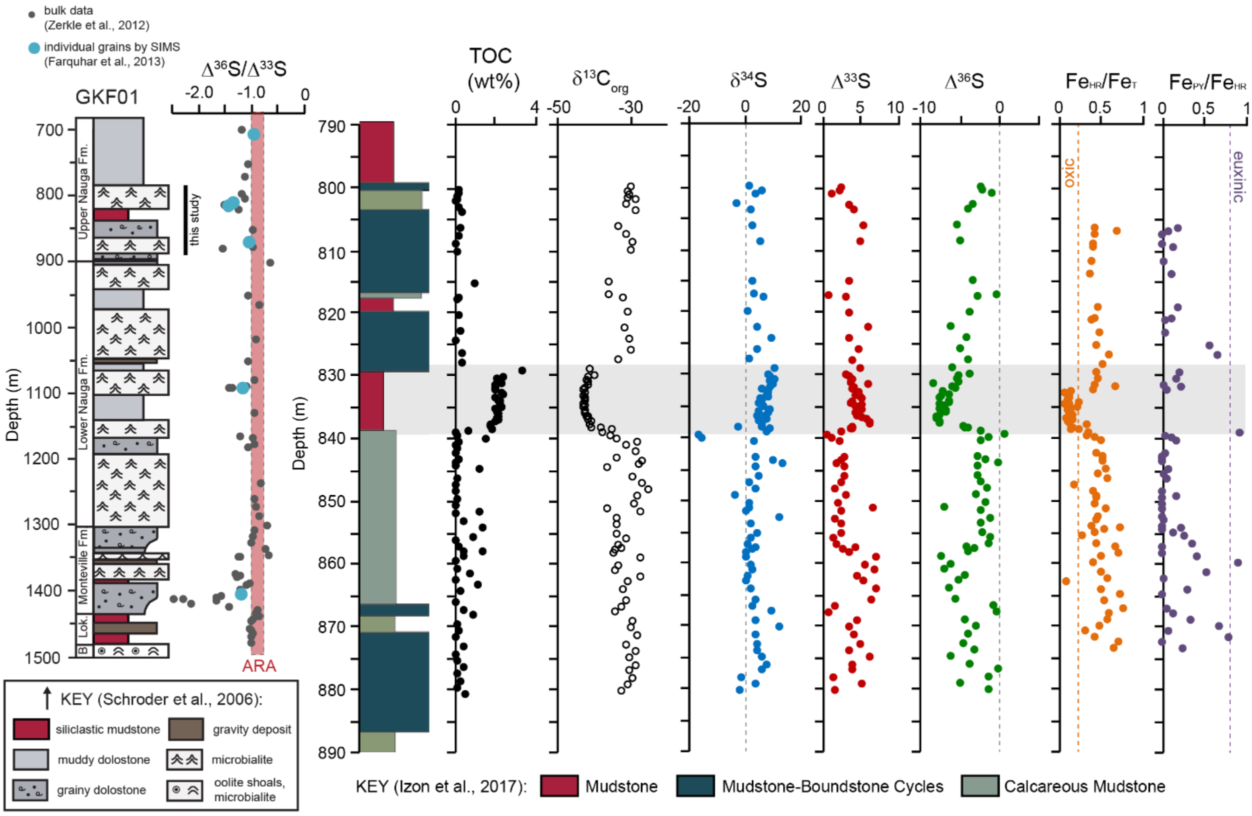

Supplementary Figure 2. Previously published geochemical data for the studied interval. The full stratigraphy for core GKF01 (from Schroder et al.[1]) is shown on the left, along with published $\Delta^{36}S/\Delta^{33}S$ data measured on bulk pyrite[2] and individual pyrite grains[3]. The red bar (ARA) is the Archean reference array (e.g., Ono et al.[4]). Also shown is published pyrite sulfur isotope data ($\delta^{34}S$, $\Delta^{33}S$ and $\Delta^{36}S$, all in ‰) and Fe speciation data (Fe_{HR}/Fe_T, and Fe_{Py}/Fe_{HR} for samples with $Fe_{HR}/Fe_T > 0.2$), alongside total organic carbon content (TOC, wt%) and organic carbon isotope data (in ‰), for the studied section, all as reported in Izon et al.[5].

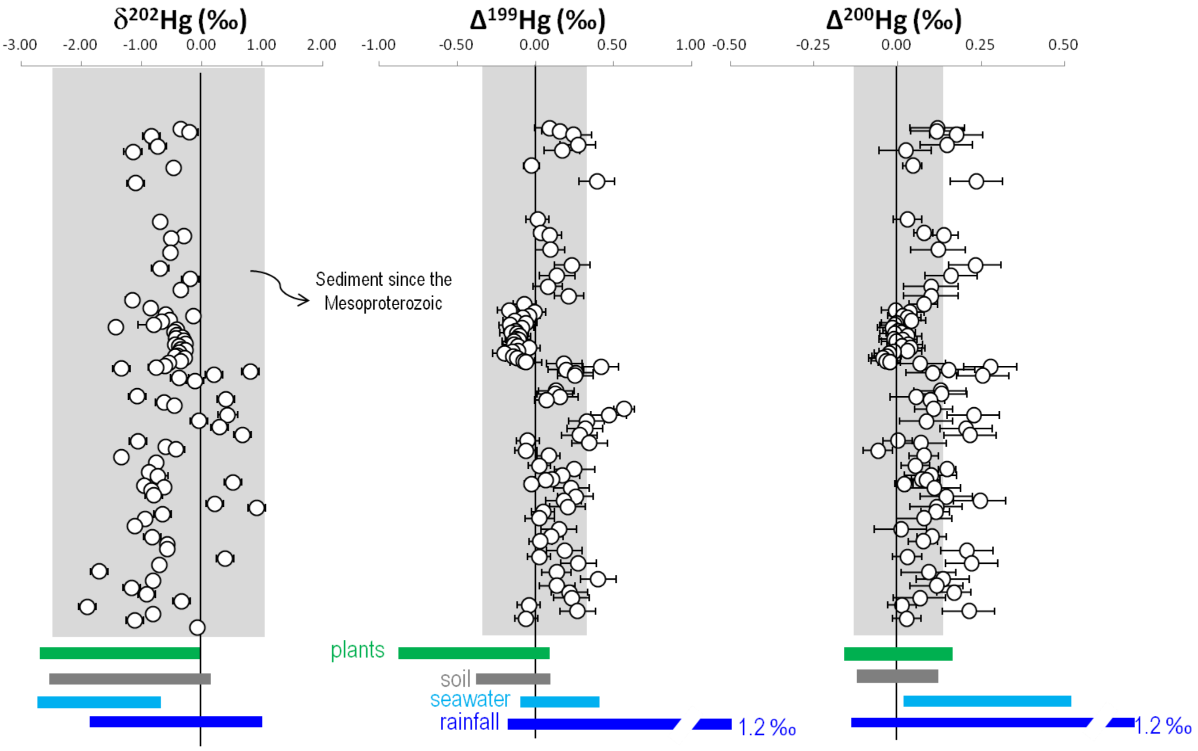

Supplementary Figure 3. Mercury isotope data for natural samples. This includes a comparison of our Hg isotope data with ranges of data previously published for plants, soil, seawater and rainfall (from Blum et al.[6] and Strok et al. [7]). Uncertainties on Hg isotope data correspond to the larger value of either the measurement uncertainty of replicate digests of MESS-2 or the uncertainty of repeated measurements of UM-Almadén, as in Figure 1.

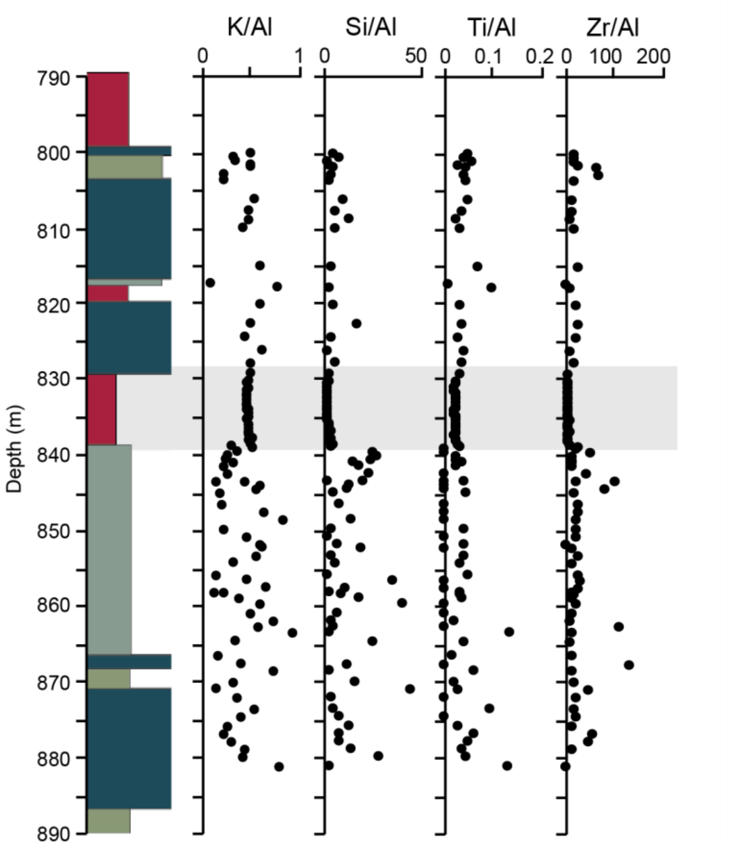

Supplementary Figure 4. Elemental ratios for the studied section. Data are listed in Supplementary Data 2 and 3.

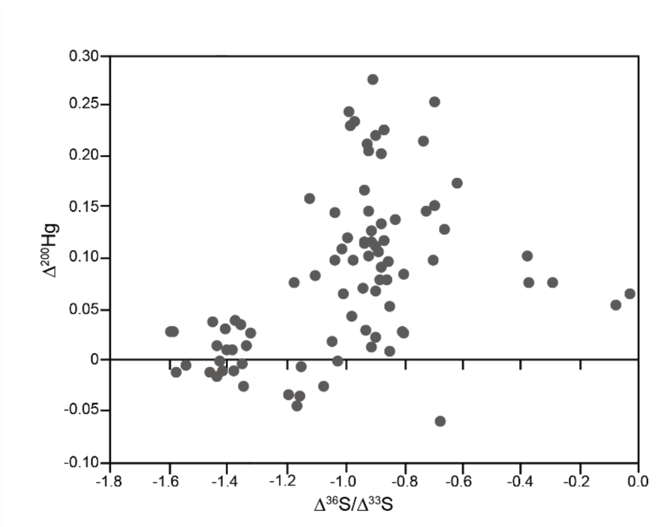

Supplementary Figure 5. Combined mercury and sulfur isotope data for the studied section. These are shown as Δ^{200}Hg (in ‰) versus $\Delta^{36}S/\Delta^{33}S$.

Supplementary Table 1. Hg isotopic compositions for UM-Almadén and MESS-2

	Preparation method	THg* ng/g	2SD ng/g	δ^{202}Hg ‰	2SD ‰	Δ^{199}Hg ‰	2SD ‰	Δ^{200}Hg ‰	2SD ‰	Δ^{201}Hg ‰	2SD ‰
MESS-2	Acid digestion	101		-1.91		-0.03		0.03		-0.06	
MESS-2	Acid digestion	95.8		-1.93		-0.04		0.03		-0.04	
MESS-2	Acid digestion	85.5		-1.87		-0.03		0.01		-0.02	
**Average**		**94.1**	**15.8**	**-1.90**	**0.06**	**-0.03**	**0.01**	**0.02**	**0.02**	**-0.04**	**0.04**
MESS-2	Combustion	98.3		-1.97		-0.02		0.02		-0.08	
MESS-2	Combustion	90.1		-1.94		-0.02		0.02		-0.02	
MESS-2	Combustion	89.2		-1.92		-0.04		0.05		-0.02	
MESS-2	Combustion	87.4		-1.89		-0.02		0.03		-0.02	
MESS-2	Combustion	95.6		-1.95		-0.04		0.02		-0.02	
MESS-2	Combustion	92.8		-1.99		-0.05		0.03		-0.05	
MESS-2	Combustion	100.1		-1.99		0.00		-0.01		-0.05	
MESS-2	Combustion	93.3		-1.90		-0.03		0.04		-0.01	
MESS-2	Combustion	88.4		-1.85		0.02		0.05		-0.04	
**Average**		**92.8**	**9.0**	**-1.93**	**0.10**	**-0.02**	**0.04**	**0.03**	**0.04**	**-0.04**	**0.05**
UM-Almadén				-0.60		-0.05		0.01		-0.04	
UM-Almadén				-0.52		0.02		0.03		-0.01	
UM-Almadén				-0.53		0.02		-0.01		-0.05	
UM-Almadén				-0.57		-0.01		0.01		-0.04	
UM-Almadén				-0.49		0.00		0.03		0.02	
UM-Almadén				-0.57		-0.05		0.00		-0.01	
UM-Almadén				-0.56		-0.07		-0.02		-0.04	
UM-Almadén				-0.53		0.00		0.05		-0.04	
UM-Almadén				-0.46		0.02		0.05		0.00	
UM-Almadén				-0.48		0.03		0.04		0.01	
UM-Almadén				-0.54		-0.01		0.01		-0.03	
UM-Almadén				-0.6		-0.08		-0.04		-0.06	
**Average**				**-0.54**	**0.09**	**-0.01**	**0.07**	**0.01**	**0.06**	**-0.02**	**0.05**

***Hg concentrations were calculated based on ^{202}Hg signals of MC-ICP-MS**

Supplementary References

1. Schroder, S., Lacassie, J. P. & Beukes, N. J. Stratigraphic and geochemical framework of the Aguoron drill cores, Transvaal Supergroup (Neoarchean-Paleoproterozoic, South Africa). *South African Journal of Geology* **109**, 23-54 (2006).
2. Zerkle, A. L., Claire, M. W., Domagal-Goldman, S. D., Farquhar, J. & Poulton, S. W. A bistable organic-rich atmosphere on the Neoarchean Earth. *Nature Geoscience* **5**, 359-363 (2012).
3. Farquhar, J. *et al.* Pathways for Neoarchean pyrite formation constrained by mass-independent sulfur isotopes. *Proceedings of the National Academy of Sciences* **110**, 17638-17643 (2013).
4. Ono, S., Wing, B., Johnston, D., Farquhar, J. & Rumble, D. Mass-dependent fractionation of quadruple stable sulfur isotope system as a new tracer of sulfur biogeochemical cycles. *Geochimica et Cosmochimica Acta* **70**, 2238-2252 (2006).
5. Izon, G. *et al.* Biological regulation of atmospheric chemistry en route to planetary oxygenation. *Proceedings of the National Academy of Sciences* **114**, 2571-2579 (2017).
6. Blum, J. D., Sherman, L. S. & Johnson, M. W. Mercury isotopes in earth and environmental sciences. *Annual Review of Earth and Planetary Sciences* **42**, 249-269 (2014).
7. Štrok, M., Baya, P. A. & Hintelmann, H. The mercury isotope composition of Arctic coastal seawater. *Comptes Rendus Geoscience* **347**, 368-376 (2015).
