# Supplementary material for: Anomalous fractionation of mercury isotopes in the Late Archean atmosphere
Source: Nat Commun. 2020 Apr 6;11:1709. doi: 10.1038/s41467-020-15495-3 (PMC7136252; doi:10.1038/s41467-020-15495-3)
Supplement: Supplementary file 3 — Description of Additional Supplementary Files [file 41467_2020_15495_MOESM3_ESM.pdf]

Description of Additional Supplementary Files

File Name: Supplementary Data 1
Description: Hg, Hg isotope, S isotope, and TOC data

File Name: Supplementary Data 2
Description: Major element data

File Name: Supplementary Data 3
Description: Trace element data
