# Supplementary material for: Anomalous fractionation of mercury isotopes in the Late Archean atmosphere
Source: Nat Commun. 2020 Apr 6;11:1709. doi: 10.1038/s41467-020-15495-3 (PMC7136252; doi:10.1038/s41467-020-15495-3)
Supplement: Supplementary file 2 — Peer Review File [file 41467_2020_15495_MOESM2_ESM.pdf]

Reviewers' comments:

Reviewer #1 (Remarks to the Author):

This paper is motivated by all the right questions, and the integration of mercury and sulfur mass independent behavior is rigorous, novel, and potentially powerful. I hope the authors can address my questions so that publication will be possible. That said, I have some serious misgivings and suggestions. The latter should be easier to address. I remain optimistic about publication overall if my concerns about facies rather than atmospheric controls on the data trends are misplaced (see below for details).

(1) I understand that the preponderance of positive Δ^{199}Hg and Δ^{200}Hg values is interesting and meaningful. That said, the data mostly scatter with relatively large errors. What is most striking, however, is the dramatic shift in all the data in the 840 to 830 interval. Here we see important negative shifts in Δ^{199}Hg and Δ^{200}Hg also seen within the S-MIF data, along with a striking tightening of the variance in Δ^{202}Hg. Substantial portions of the paper focus on the significance of this portion of the section as the 'haze interval.' My problem is that it corresponds exactly with a distinct lithologic unit, with the data shifting in exact sympathy with the lithologic changes. Interpretations of regional or global processes, such as atmospheric conditions, defined by a geochemical record confined to a specific lithology raise many flags, and I am really worried that the trends are telling us more about facies dependence than atmospheric properties and processes. Or do the authors think the shale was caused by atmospheric processes and related organic delivery? Surely not. If the shale window is, instead, a special lithologic window that captures and preserves a longer-lived condition, than the intervals above and below have far less atmospheric relevance. In short, the data/lithologic correspondence demands more attention.
(2) Lines 180-181: We are told that the Hg-MIF changes are not confined to this siliciclastic shale unit. In truth, the dramatic negative shifts and tightening of the data are confined exactly to this interval. This is a worry.
(3) It's a worry because these depths are described as the organic-rich interval, and given relationships describe by the authors between Hg isotope behavior and organic interactions, I wonder if the observations aren't an artifact of those enrichments and may have less to do with atmospheric processes and more to do lithologic artifacts. Given the dramatic Hg concentration enrichments in this interval, against consistent Hg/TOC ratios throughout the section, TOC must be dramatically higher in the 840-830 interval compared to the over- and underlying strata.
(4) Inclusion of a TOC profile in Fig. 1 would be very helpful in this regard.
(5) Similarly, my guess is that the 840-830 S-MIF shift corresponds with a shale that is hugely enriched in pyrite relative to the sediments above and below. Could this not lead to an S isotope artifact? We should be told (1) that the S data are from pyrite, assuming they are, and (2) what the stratigraphic trends for pyrite abundance are. If these details are in the supplement, they probably shouldn't be — or at least they should be called out more effectively and discussed briefly in the primary text. There is something interesting happening in the interval, but I'm just not sure what and

whether it reflects atmospheric change rather than facies dependence. I am happy to be missing something obvious, but other readers will raise the same questions. The authors must address these concerns more aggressively and convincingly.

(6) In general, we need far more information about the lithologies and their variations. The details in the stratigraphic column of Fig. 1 are extremely sparse to the point of being pretty useless. There are references to dolostones not shown in the column, there is likelihood of huge differences in sedimentation rates (including relative starvation for the shale interval) that could affect the other data, etc.

(7) Line 59: "Gaseous Hg(0) can be removed from the atmosphere either through: (1) transformation to gaseous oxidized Hg(II) species, which are particle reactive…" Line 231-234: "the slightly negative to zero values of Δ^{199}Hg and Δ^{200}Hg in the interval from 829.22-838.78 m depth also support the increased importance of atmospheric Hg(0) as a source of Hg to the oceans." I am confused. If an isotope effect is related to transformation of Hg(0) to Hg(II), and Hg(II) is particle reactive and thus efficiently scavenged, why don't we see a corresponding increase in Hg/TOC through this interval? I am missing something. More generally, shouldn't any major isotopic shift be expressed in [Hg] and Hg/TOC? In other words, Hg has a high affinity for OM, and changes in particle reactivity with valence shifts are associated with isotope effects, so shouldn't an enrichment in Hg relative to TOC occur in concert with isotopic shifts?

(8) The paragraph starting with line 113: There are lot of details here about Hg cycling and related isotope effects. They are critical to the authors' story but are hard to follow. A schematic figure outlining these effects would help.

(9) Lines 194-195: Again is it a common mechanism (as in atmospheric) or similar responses to facies controls?

(10) Line 214: Just because another paper asserts AOM as a control, it doesn't mean that volcanic S inputs weren't instead the dominant factor. In other words, if the authors cite another paper for vital validation of their argument, they probably need to defend their adoption of those arguments — given the importance of the assumption in developing their story.

(11) Line 227: Organic matter in the atmosphere? Probably "organic compounds" would be better.

Reviewer #2 (Remarks to the Author):

My comments are on the attached pdf. In my assessment publication of a revised version of this work by Nature Communications is justified. Not only are the data a new type and used in a new way, but they also reveal a surprising novel relationship (different from modern data and with systematic variation) that provides new insight into one of the key questions in understanding late-Archean atmospheric evolution and events. The current paradigm of Archean atmospheric events is strongly linked to feedbacks related to oxygen and methane and the role of organic haze aerosols in modulating UV transparency and in impacting atmospheric reaction networks. Arguments for late Archean atmospheric events are strong, hinging on relationships between D36/D33 and d13C, but not unquestioned and it is vital that they be tested. This study brings a new (but well-established for the modern) system to bear on this issue. The study documents a relationship between mercury isotopes that is different than that seen today and in the more recent geologic record. Moreover, this relationship varies in concert with the prior observations of D36S/D33S and d13C. I view this as a nail in the case for the atmospheric event -related origin of these signals and by extension, for the connection to methane. This prior hypothesis was

presented originally in Nature Geoscience and has been a focus of intense interest and debate. Another aspect of the Hg data presented here are the differences between the relationships seen in the Archean dataset and the existing global dataset of sedimentary Hg data. These differences point to different source chemistry with an atmospheric connection. Due to its nature, this will become a focus of active research to come. So I strongly believe that this study deserves publication in a journal such as the Nature family journals. I would place it high in that group - thinking even Nature.

These points made, there are a number of comments that I would like the authors to address and consider. They are marked in the attached manuscript. Some of this may warrant significant changes, but this I see as at the author's discretion. I (James Farquhar) am available for discussion of my comments and look forward to seeing this study in print.

Reviewer #3 (Remarks to the Author):

Zerkle et al. present an interesting new dataset featuring both mercury and sulphur isotopes, and attempt to use these geochemical parameters to investigate atmospheric developments shortly prior to the GOE. I have not seen mercury isotopes used in this way before, so the idea is certainly novel and interesting, and has the insurance of using them alongside a much more established proxy of Archean atmospheric studies in the sulphur MIF.

However, I feel that the interpretations presented by the authors are not always as strong as they suggest, and in some of the claims are unsubstantiated. Whilst they do acknowledge that both the mercury and sulphur cycles are complex and that there is much that we do not know about them (especially in the Archean, where both cycles were likely rather different), they rule out some of the possible controls on the mercury isotopes a little too easily. I don’t think that they have as strong a case for ruling out volcanism and runoff as impactors as they claim. Also, there appears to be an inherent assumption made that because the perturbations in sulphur and mercury occur at the same level (829.22–838.78m depth), they must be directly related to the same process. This might be the case, but it doesn’t have to be. I think that more thought about the non-atmospheric alternatives for the observed geochemical perturbations is needed, alongside additional data that can help rule out some of these other controls more conclusively, in order to strengthen the arguments presented here. Therefore, I cannot recommend that this manuscript be accepted by Nature Communications at this time.

Major Comments:

The authors state that the change in Hg MIF is not entirely confined to the mudstone interval. Looking at figure 1, I am not at all convinced by this statement. At best, only the very bottom and topmost samples occur just below and above, respectively, the mudstone, and they record the decline into and recovery from the excursions. Also can the authors be absolutely certain that there could not be some movement of mercury at the interface of the two different lithologies. Frustratingly, the two other mudstone intervals in the section have only one data point between the two of them, so it is impossible to judge what signal is recorded in the other mud units. If I’m wrong about this, then a zoomed-in stratigraphic figure showing just the main mudstone unit and a meter either side might help make this clear.

The authors are not entirely correct to rule out the occurrence of volcanism on the absence of a Hg/TOC signal. Yes, many Phanerozoic igneous provinces have been linked to Hg/TOC peaks, but it has been shown that not all of them generate such a peak. Moreover, even for mega volcanic events that do cause Hg/TOC enrichments, the spikes do not always happen throughout the entire history of volcanism, and nor does every sedimentary record deposited at that time manifestly record them (see Percival et al., 2018, American Journal of Science; and Grasby et al., 2019, Earth

Science Reviews). Of course, one could argue that if there isn't sufficient volcanic Hg arriving at this location to cause a Hg/TOC peak, how could there be enough to affect the Hg MIF? It's tricky, but one could envisage a scenario where TOC rises independently of Hg (note the change in lithology to mudstones which could allow this), in which case excess volcanic Hg devoid of MIF could arrive and change the sedimentary MIF composition, but the extra TOC cancel out any Hg/TOC peak. A slightly convoluted scenario, but not an impossible one.

However, I actually think that runoff of Hg is the more plausible alternative. The authors do mention runoff, but then rule it out on the basis that the terrestrial biosphere did not exist in the Precambrian to provide negative MIF from land. However, geogenic Hg is also thought to be devoid of MIF in both odd and even Hg isotopes (see Blum et al's 2014 review), and this could still have been present in terrestrial runoff in the Archean. A recent paper by Zheng et al. (2018, PNAS) also suggested that negative MIF of Hg could also arise through development of euxinic conditions, this should be mentioned as well.

Because a lithological change does seem to be a feature of most (if not all) of the stratigraphic level recording the Hg and S perturbations, such local perturbations should be considered in more detail. The authors already mention Fe speciation as evidence for redox conditions during the core. I would suggest a new figure presenting this data, along with any further S data (S contents? d34S?) that can relate redox conditions and any connection between redox and Hg. I would also strongly suggest presenting detrital proxy data (e.g., Ti/Al, Si/Al, Zr/Rb) in this figure to confirm what the role of terrestrial runoff (if any) might be. It is a pity that there is only one stratigraphic record presented here; a second record might have greatly strengthened the case against local environmental influences on these proxies. However, given the difficulties in even finding two demonstrably stratigraphically correlated Archean records, never mind the time-consuming nature of the analyses, incorporating a second site record is clearly beyond the scope of this work. But further analyses to highlight redox/runoff conditions would be very important.

Finally, even if the authors are correct in their assessment that these perturbations are related to atmospheric changes, why do they have to be connected to an organic haze, and not a slight increase in atmospheric oxygen (leading to more ozone). This has been related to changes in S MIF previously, and 'whiffs' of oxygen have long been thought to occur in the latest Archean. Maybe that doesn't help explain the change in Hg MIF, but as noted above, there are other ways to bring in Hg devoid of MIF besides atmospheric fluctuations.

In summary, more detailed consideration of possible alternative controls on these perturbations, especially the Hg MIF, is needed before this manuscript can be accepted. Additional geochemical data highlighting the potential signals of changes in redox and terrestrial runoff should be presented as part of this. Because this represents significant changes to the manuscript, I cannot currently recommend it for publication in its current form.

Minor comments:

L. 34: In this sentence, 'we' refers to 7 billion humans. Throughout the rest of the manuscript, 'we' refers to the authors. I suggest a rephrase in this context.

L. 52: Some would define the atmosphere as part of the Earth's surface, in which case the transition of chemical species from the atmosphere to the Earth's surface doesn't work as a statement. I assume that the authors are referring to the aquatic and terrestrial realms when they talk about the Earth's surface. But this needs to be made clearer.

L. 64: Change 'could' to 'would'. I think that one can fairly confidently state that Hg biogeochemical cycling would definitely have been rather different in the Precambrian.

L. 74: Include uptake by the terrestrial biosphere should be included here. Yes, I know that it is not applicable for Precambrian, but it should be stated for completeness. It can then be subsequently ruled out as a process of any importance in the context of this study.

L. 96–101: I suspect that the conclusion drawn here is correct, that the increased Hg concentration is related to increased burial efficiency by the additional organic carbon. But a note of caution should be issued, a number of the samples have rather low TOC contents. General protocol at the moment is to not use TOC values <0.2 wt% to normalize Hg against, as such low values carry comparatively large %uncertainties which consequently inflate the uncertainty in any Hg/TOC ratios. At the very least, the authors need to acknowledge this issue with some of the samples, and potentially remove the Hg/TOC ratios associated with those TOC values from the figure. As I say, in this case I don't think that it effects the author's conclusions, but it should be done to move the study in line with current practice.

L. 131–133: Somewhere here the study of Zheng et al. (2018, PNAS) about the control of photic zone euxinia on Hg odd isotope MIF needs to be mentioned.

L. 159: The exact mechanism of depositing Hg(0) should be clarified. Direct take up by vegetation is not an option in the Precambrian, and depositing a gas (the chief form of Hg0 in the atmosphere) is rather counterintuitive concept.

L. 180–182: As I mentioned above, I am not completely convinced by this statement that the Hg MIF extends to beyond the mudstone unit. At best, only the topmost and bottommost samples do, and there are background samples that register no/negative MIF anyway. Also the other lithologies do feature mud-rich strata as well according to the key in figure 1. I suggest that a new figure focusing on the mudstone interval and a few meters either side (possibly with a more detailed lithological column) would help address this.

L. 183–186: Contribution of Hg from runoff of terrestrial vegetation and organic matter would indeed have presumably been negligible during the Precambrian. But what about geogenic Hg in runoff. This is also (albeit on the basis of a limited number of studies) devoid of MIF, so an increase in terrestrial runoff might also shift the sedimentary composition of MIF towards 0, and also potentially increase the clay influx to this marine setting that would be reflected by the observed change in lithology.

L. 207: Volcanic emissions with no Hg MIF are based on studies of a single volcanic system, which is an arc system. There is no guarantee that this is representative of volcanism as a whole today, let alone in the Archean when plate tectonics was rather different. Of course, these are the data that we have, so we have to make that assumption for now, but at the very least, the authors should be clear about the limitations of this assumption.

L. 207–208: This is not entirely correct. Some Phanerozoic Large Igneous Provinces have been claimed as recorded by Hg/TOC peaks, but this is not the case for all such volcanic events, and even when Hg/TOC peaks are observed, most case studies show that not all sedimentary records coeval with those eruptions will show the enrichments. So whilst one should certainly not infer a volcanic influence just on the basis of this dataset, neither can one rule it out because there is not a Hg/TOC spike.

L. 210–214: Redox conditions are discussed here. It would be good to put these into a figure (along with runoff proxies such as Ti/Al, Si/Al, Zr/Rb) so that stratigraphic trends can be compared to the Hg- and S-isotope data. This can then be used to clarify whether or not there is any relationship between these changes in MIF and local environmental conditions.

Reviewer #1 comments and responses:

This paper is motivated by all the right questions, and the integration of mercury and sulfur mass independent behavior is rigorous, novel, and potentially powerful. I hope the authors can address my questions so that publication will be possible. That said, I have some serious misgivings and suggestions. The latter should be easier to address. I remain optimistic about publication overall if my concerns about facies rather than atmospheric controls on the data trends are misplaced (see below for details).

We thank the reviewer for their support, and for the opportunity to further examine other potential controls on the Hg isotope data we present. We maintain that one of the most exciting aspects of this study is the identification *for the first time* of large magnitude Hg-MIF in Archean-age sediments. Further, the magnitude of the Δ^{199}Hg and Δ^{200}Hg values we measure (> 0.5‰ and 0.25‰, respectively) are larger than any Hg-MIF values reported from younger sediments (e.g., Figure 3), and show very different trends than any previously published data (e.g., Figure S4). Since even-numbered Hg-MIF in particular (Δ^{200}Hg) is only known to be produced in the atmosphere, these data **fundamentally require an atmospheric control**. We have rewritten the discussion and restructured the manuscript to more clearly highlight the importance of this critical aspect of the study.

The majority of (this and other) reviewers' concerns seem to be related to our interpretation of the change in Hg-MIF values across the organic-rich interval. We have significantly rewritten the manuscript to address the more carefully consider other processes that could be responsible for controlling these changes (including facies changes), and have added a significant amount of (new and previously published) data which collectively most strongly support a role for atmospheric changes and volcanic emissions in producing these specific changes.

(1) I understand that the preponderance of positive Δ^{199}Hg and Δ^{200}Hg values is interesting and meaningful. That said, the data mostly scatter with relatively large errors.

The Hg-MIF signals that we document here are fully resolvable, and the errors are standard for this type of analysis. The Δ^{199}Hg and Δ^{200}Hg values in this study range from -0.2 to +0.57‰ and from -0.06 to +0.28, respectively, which confirm significant MIF of Hg isotopes, considering the analyatical errors were 0.07‰ (2σ) for Δ^{199}Hg and 0.06‰ (2σ) for Δ^{200}Hg in this study (as detailed in the SI).

What is most striking, however, is the dramatic shift in all the data in the 840 to 830 interval. Here we see important negative shifts Δ^{199}Hg and Δ^{200}Hg also seen within the S-MIF data, along with a striking tightening of the variance in Δ^{202}Hg. Substantial portions of the paper focus on the significance of this portion of the section as the 'haze interval.' My problem is that it corresponds exactly with a distinct lithologic unit, with the data shifting in exact sympathy with the lithologic changes. Interpretations of regional or global processes, such as atmospheric conditions, defined by a geochemical record confined to a specific lithology raise many flags, and I am really worried that the trends are telling us more about facies dependence than atmospheric properties and processes. Or do the authors think the shale was caused by atmospheric processes and related organic delivery? Surely not. If the shale window is, instead, a

special lithologic window that captures and preserves a longer-lived condition, than the intervals above and below have far less atmospheric relevance. In short, the data/lithologic correspondence demands more attention.

As above, the change in Hg-MIF across the 840-830 m interval is striking, but we maintain that the identification *for the first time* of large magnitude Hg-MIF in Archean-age sediment is actually the most compelling aspect of this study. In addition, the generation of the even-numbered Hg-MIF fundamentally requires an atmospheric control. While interesting, the change in Hg-MIF across the organic-rich mudstone interval is of secondary importance.

We note that there is no direct lithologic control on the production of Hg-MIF, particularly for Δ^{200}Hg, which is only produced in the atmosphere. However, we acknowledge that a change in sedimentary Hg-MIF values could represent a change in Hg sources or burial. We have rewritten the discussion to more thoroughly examine all the possibilities that could have led to the shifts in Δ^{199}Hg and Δ^{200}Hg values across this interval (Lines 208-260), which include:

(1) Enhanced sequestration of atmospheric Hg(0) to the sediments by thiols and sulfide that were enriched in the surface ocean as a result of photic zone euxinia (e.g., Zheng et al., 2018).
(2) Enhanced terrestrial input of Hg with no MIF signals, which also may have shifted the Δ^{199}Hg and Δ^{200}Hg to near-zero values.
(3) Enhanced volcanic release of Hg with no MIF signals, which could have shifted the Δ^{199}Hg and Δ^{200}Hg to near-zero values.
(4) A change in atmospheric transparency or atmospheric chemistry, leading to changes in the production of Hg-MIF in the atmosphere or delivery of Hg-MIF to the sediments.

We have added water column paleoredox data (Fe speciation and total S) for this section that argue against enhanced sulfide scavenging (scenario 1) (Figures 2S, S2), and terrestrial proxy data (elemental ratios) that argue against a significant change in terrestrial influx (scenario 2) (Figure S4). We have expanded our consideration of a volcanic influence on these trends (scenario 3), following the advice of R3 below, and interpret very high Hg concentrations and Hg/TOC ratios as indicative of volcanic Hg flux in the upper part of this section. However, REE data from lower in the mudstone interval argue against volcanism as the primary driver throughout this interval (as discussed in Lines 239-249). Given the lack of evidence for enhanced euxinia or terrestrial input, and only discrete evidence for enhanced volcanism, we suggest that the changes in Hg-MIF within this interval were largely due to a change in atmospheric chemistry (scenario 4), and the negative Δ^{199}Hg values at the bottom of this interval (Figures 1) support the increased importance of atmospheric Hg(0) as a source of Hg to the oceans.

A change in atmospheric chemistry is further supported by previously published geochemical data from this section, including S-MIF values preserved in pyrite and δ^{13}C of organic carbon. The interpretation of these data as representing a global atmospheric haze event is based on a significant body of previously published, multi-proxy data, from Late Archean sediments from 5 cores on 2 continents. These previously published S-MIF records have been shown to be independent of facies controls (Zerkle et al., 2012, Nature Geo; Izon et al., 2015, EPSL; 2017, PNAS) and are conserved at the grain scale (Farquhar et al., 2014, PNAS). We have further expanded our discussion of this previously published data (and supporting atmospheric models)

in the manuscript (Lines 261-285), and added a supplementary figure showing previously published records from this section and throughout the full GKF01 core (Figure S2).

(2) Lines 180-181: We are told that the Hg-MIF changes are not confined to this siliciclastic shale unit. In truth, the dramatic negative shifts and tightening of the data are confined exactly to this interval. This is a worry.

As above, we have rewritten the manuscript to acknowledge that the changes in Hg concentration and Hg-MIF appear to correspond to a change in lithology; however, we argue that this follows from the strong affinity of Hg for organic matter (potentially sourced from the organic haze) which does not produce the signal but does enhance the delivery of this signal to the sediments.

(3) It's a worry because these depths are described as the organic-rich interval, and given relationships describe by the authors between Hg isotope behavior and organic interactions, I wonder if the observations aren't an artifact of those enrichments and may have less to do with atmospheric processes and more to do lithologic artifacts. Given the dramatic Hg concentration enrichments in this interval, against consistent Hg/TOC ratios throughout the section, TOC must be dramatically higher in the 840-830 interval compared to the over- and underlying strata.

This is correct – TOC increases across the same interval (we have added this dataset to Figure 1). However, the process of organic matter scavenging alone can not produce or change Hg-MIF, particularly for Δ^{200}Hg, which is only formed in the atmosphere. Therefore, organic scavenging is not a direct mechanism for changing Hg-MIF values in sediments. See further discussion in above.

(4) Inclusion of a TOC profile in Fig. 1 would be very helpful in this regard.

We have added a TOC profile to Figure 1, as requested.

(5) Similarly, my guess is that the 840-830 S-MIF shift corresponds with a shale that is hugely enriched in pyrite relative to the sediments above and below. Could this not lead to an S isotope artifact? We should be told (1) that the S data are from pyrite, assuming they are, and (2) what the stratigraphic trends for pyrite abundance are. If these details are in the supplement, they probably shouldn't be — or at least they should be called out more effectively and discussed briefly in the primary text. There is something interesting happening in the interval, but I'm just not sure what and whether it reflects atmospheric change rather than facies dependence. I am happy to be missing something obvious, but other readers will raise the same questions. The authors must address these concerns more aggressively and convincingly.

Yes, the previously published S isotope data are from sedimentary pyrite, we have clarified this in the manuscript (Line 263). As in response to point (1) above, enhanced sequestration of atmospheric Hg(0) to the sediments by thiols and sulfide that were enriched in the surface ocean as a result of photic zone euxinia could potentially cause a decrease in Hg-MIF. We have added previously published Fe speciation and S abundance data that argue against this mechanism as controlling changes in Hg-MIF across the shale interval (Figures 2S, S2). In contrast, a change in

pyrite burial would have no effect on S-MIF, so could not explain the change in $\Delta^{36}S/\Delta^{33}S$ ratios. Alternative hypotheses for the changes in S-MIF signals across this interval are presented in Izon et al. (2017, PNAS) – it is beyond the scope of this study to fully rehash these arguments. Instead, we examine how the preferred (previously peer-reviewed and published) interpretation of the S-MIF data can support or refute hypotheses the correlative changes in Hg-MIF that are the focus of this study.

(6) In general, we need far more information about the lithologies and their variations. The details in the stratigraphic column of Fig. 1 are extremely sparse to the point of being pretty useless. There are references to dolostones not shown in the column, there is likelihood of huge differences in sedimentation rates (including relative starvation for the shale interval) that could affect the other data, etc.

These are relatively unremarkable Precambrian rocks (which is why they are excellent for geochemical analyses!) so there is not much else to say about them. We have included the most detailed stratigraphic and lithologic descriptions here, available from the Agouron-Griqualand Paleoproterozoic Drilling Project (AGPDP) Online Database http://general.uj.ac.za/agouron/index.aspx, and published in Izon et al. (2017, *PNAS*). In addition, this interval encompasses only 100 meters of a 1.5 km drillcore, thus there is not even a specific depositional model for the associated facies change that happens over ~10 meters. We must therefore rely on general depositional models for the basin as a whole, as published in Schroder et al. (2009) and described in lines 90-100. Further, we make no reference to dolostones in this interval, although they are found throughout the greater GKF01 core (Figure S2).

(7) Line 59: "Gaseous Hg(0) can be removed from the atmosphere either through: (1) transformation to gaseous oxidized Hg(II) species, which are particle reactive…" Line 231-234: "the slightly negative to zero values of Δ^{199}Hg and Δ^{200}Hg in the interval from 829.22-838.78 m depth also support the increased importance of atmospheric Hg(0) as a source of Hg to the oceans." I am confused. If an isotope effect is related to transformation of Hg(0) to Hg(II), and Hg(II) is particle reactive and thus efficiently scavenged, why don't we see a corresponding increase in Hg/TOC through this interval? I am missing something. More generally, shouldn't any major isotopic shift be expressed in [Hg] and Hg/TOC? In other words, Hg has a high affinity for OM, and changes in particle reactivity with valence shifts are associated with isotope effects, so shouldn't an enrichment in Hg relative to TOC occur in concert with isotopic shifts?

As above, scavenging by organic matter does not directly produce Hg-MIF, it just acts as a carrier of the Hg-MIF signal from the surface ocean into the sediments. In this case we are calling on a change in the source of this Hg-MIF. We suggest that the positive Δ^{199}Hg and Δ^{200}Hg values preserved throughout the majority of this section represent "normal" deposition of particle-reactive Hg(II) species that underwent intense photo-oxidation and reduction in the absence of an ozone layer. We further suggest that the negative Δ^{199}Hg values in the shale interval represent an unusual atmospheric state where Hg(0) was the main source of Hg to the oceans and sediments. We have further clarified this distinction throughout the discussion, and created a new schematic (Figure 4) to illustrate the relevant processes.

(8) The paragraph starting with line 113: There are lots of details here about Hg cycling and related isotope effects. They are critical to the authors' story but are hard to follow. A schematic figure outlining these effects would help.

We have added the following Figure 4 to the manuscript, which illustrates these processes for the background Archean atmosphere (Figure 4A) and for a hazy period (Figure 4B).

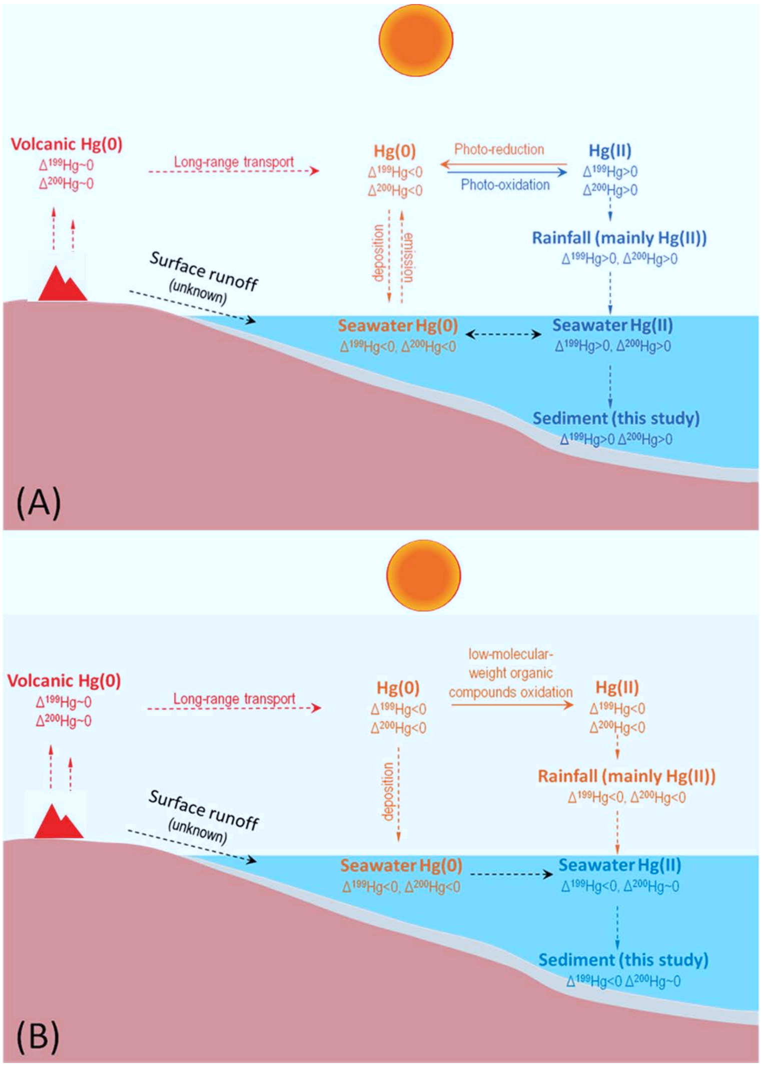

Figure 4. Proposed scenarios for producing the observed trends in Hg-MIF, for the background Archean atmosphere (A), and in the presence of a hydrocarbon haze (B).

(9) Lines 194-195: Again is it a common mechanism (as in atmospheric) or similar responses to facies controls?

See responses above.

(10) Line 214: Just because another paper asserts AOM as a control, it doesn't mean that volcanic S inputs weren't instead the dominant factor. In other words, if the authors cite another paper for vital validation of their argument, they probably need to defend their adoption of those arguments — given the importance of the assumption in developing their story.

This statement was not particularly important to our argument. Regardless, as above, we have rewritten this discussion and added further consideration of the role of volcanic S inputs in controlling the Hg-MIF trends (Lines 236-249).

(11) Line 227: Organic matter in the atmosphere? Probably "organic compounds" would be better.

We have changed the sentence accordingly.

Reviewer #2 comments and responses:

My comments are on the attached pdf. In my assessment publication of a revised version of this work by Nature Communications is justified. Not only are the data a new type and used in a new way, but they also reveal a surprising novel relationship (different from modern data and with systematic variation) that provides new insight into one of the key questions in understanding late-Archean atmospheric evolution and events. The current paradigm of Archean atmospheric events is strongly linked to feedbacks related to oxygen and methane and the role of organic haze aerosols in modulating UV transparency and in impacting atmospheric reaction networks. Arguments for late Archean atmospheric events are strong, hinging on relationships between D36/D33 and d13C, but not unquestioned and it is vital that they be tested. This study brings a new (but well-established for the modern) system to bear on this issue. The study documents a relationship between mercury isotopes that is different than that seen today and in the more recent geologic record. Moreover, this relationship varies in concert with the prior observations of D36S/D33S and d13C. I view this as a nail in the case for the atmospheric event -related origin of these signals and by extension, for the connection to methane. This prior hypothesis was presented originally in Nature Geoscience and has been a focus of intense interest and debate. Another aspect of the Hg data presented here are the differences between the relationships seen in the Archean dataset and the existing global dataset of sedimentary Hg data. These differences point to different source chemistry with an atmospheric connection. Due to its nature, this will become a focus of active research to come. So I strongly believe that this study deserves publication in a journal such as the Nature family journals. I would place it high in that group - thinking even Nature.

These points made, there are a number of comments that I would like the authors to address and consider. They are marked in the attached manuscript. Some of this may warrant significant changes, but this I see as at the author's discretion. I (James Farquhar) am available for discussion of my comments and look forward to seeing this study in print.

We thank Prof Farquhar for his support and excellent suggestions. Minor suggestions from the pdf were all incorporated into the text where still relevant. We provide responses to the main points here:

Check the later comment at line 156 that relates to the normalization of Hg isotopes and whether the MI reaction(s) signature)(s) can be distinguished from the MD reaction signature. I suspect you have a smoking gun type signature that may be different from what is observed today. If so, I would revisit the point you make in the abstract and in the paper.

We thank the reviewer for this suggestion, and address it more fully below.

I think the emphasis should be even more strongly on Hg. In other words, I think the Hg tells something very new that the S and C could not. And I do not think the S- and C are necessarily the backup for the Hg, but that the support goes the other way Hg to S and C.

We agree with the reviewer, and have significantly revised the manuscript to more fully emphasize the importance of the Hg-MIF data. However, we have had to expand our discussion

of the previously published evidence and interpretations of the corresponding S-MIF data, following requests by R1.

I do not think the paper directly constrains the composition of the pre GOE atmosphere, but I think it directly constrains a change that occurred to the pre GOE atmosphere and addresses arguments that have been raised against an atmospheric origin for changes in D33S/D36S. I suspect it pins the reaction to changes in UV available for to drive the chemistry and therefore connections to methane and haze.

We thank the reviewer for his support, and have revised the abstract accordingly.

Consider showing the range of present d202 Hg range in figure (Figure 1). Similarly, consider including this information (ranges of Hg isotope values for various reservoirs) in graphical form in some way on Figure 1.

We would prefer to focus on our data in Figure 1; however we have added the following figure, illustrating these ranges, into the SI (new Figure S3).

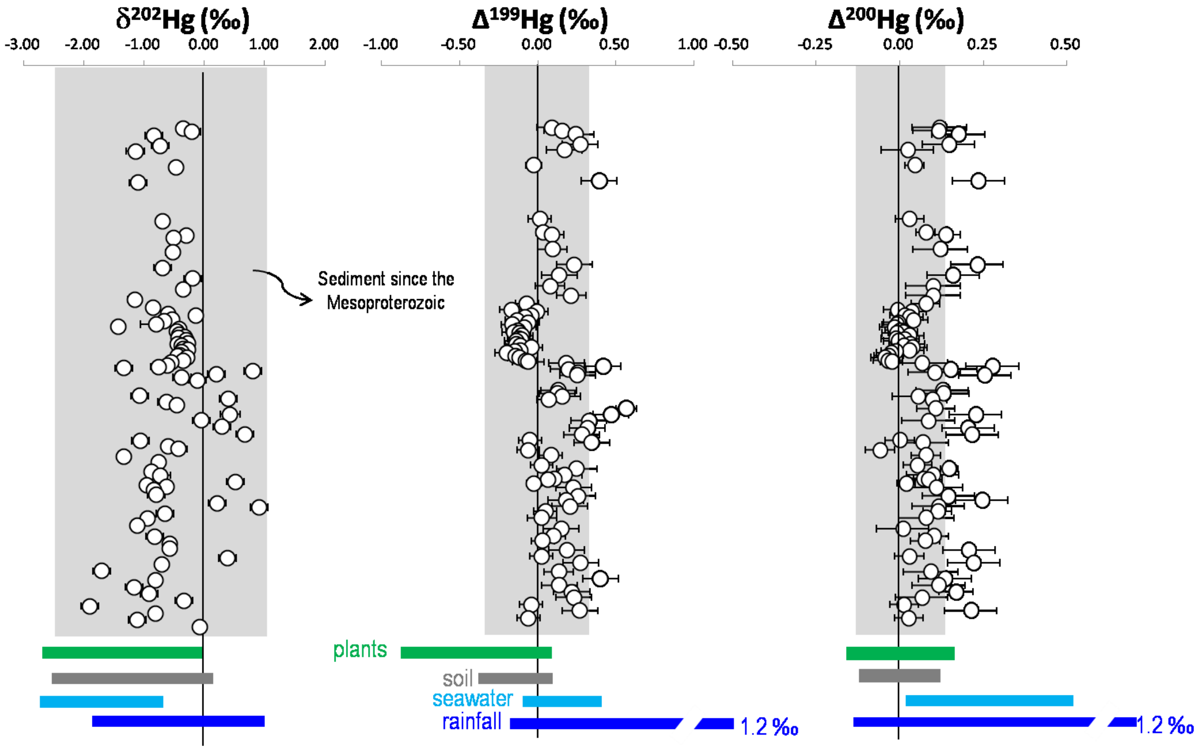

Figure S3. Comparison of our Hg isotope data with ranges of data previously published for plants, soil, seawater and rainfall (from Blum, 2014; Strok, 2015).

A point and a question (wrt the negative Hg-MIF values). Point: are they statistically resolved from zero. If so are the values MIF? This is critical for later interpretations you make.

The Δ199Hg values within the interval from ~830-840 m depth range from -0.2 to -0.01‰, which are statistically resolved from zero, considering the analytical error was 0.07‰ (2σ) for

$\Delta^{199}Hg$ in this study. And yes, these values are indeed Hg-MIF. When $\Delta^{199}Hg$ values are lower than -0.07‰, the samples are consider to have negative MIF.

Are these sediments and their respective depths of deposition sufficient to draw a parallel with the modern ocean sediment statement made above? I suspect not, but pose the question for consideration. It seems like the positive and negative are flipped.

Unfortunately no, this is not the case for modern ocean sediments with similar depth of deposition.

Just reading through and looking at the data. There is one thing I would like the authors to check.

Is it possible to deconvolve the MD effect from an MI effect(s)? I think it will be possible if there is one MI effect and it might be possible to test whether (if there is one effect) it is systematic with respect to one of the isotopes. I did an exercise that I am not sure if it is correct (renormalized the delta values to each of the other 4 Hg isotopes). It appears to me that there is a strong linearity when the isotopes are normalized to 202Hg rather than any of the other 4 isotopes (at least for the top part of the core). Can the authors check this observation for the whole dataset? Does this same observation apply to datasets for the modern?

We thank Prof Farquhar for this excellent suggestion. Below we plot the data from our study and compare the results with that of modern sediment and soil samples measured in the same lab. As shown in the left side of the figure below, for both NIST-3133 and UM-Almadén Hg standard solutions, the Hg isotope ratios ($^{199}Hg/^{198}Hg$, ^{200}Hg/198Hg, $^{201}Hg/^{198}Hg$, $^{202}Hg/^{198}Hg$, recommended by Blum and Bergquist, 2007) were linearly correlated with each other during analysis. These correlation lines reflect mass-dependent fractionation (MDF) lines because NIST-3133 and UM-Almadén Hg standard solutions have been proven to have no mass-independent fractionation (MIF), according to Blum and Bergquist (2007). Theoretically, samples with positive MIF should be beyond the MDF lines, whereas samples with negative MIF should be underneath the MDF lines. This is indeed the case. As shown in the following figures, Modern sea sediments and the other samples in our study, which show slightly positive $\Delta^{199}Hg$, $\Delta^{200}Hg$, $\Delta^{201}Hg$ values, are beyond the MDF lines. Modern soil samples and the 830-840m samples in our study, which show slightly negative $\Delta^{199}Hg$, $\Delta^{200}Hg$, $\Delta^{201}Hg$ values, are located bellow the MDF lines.

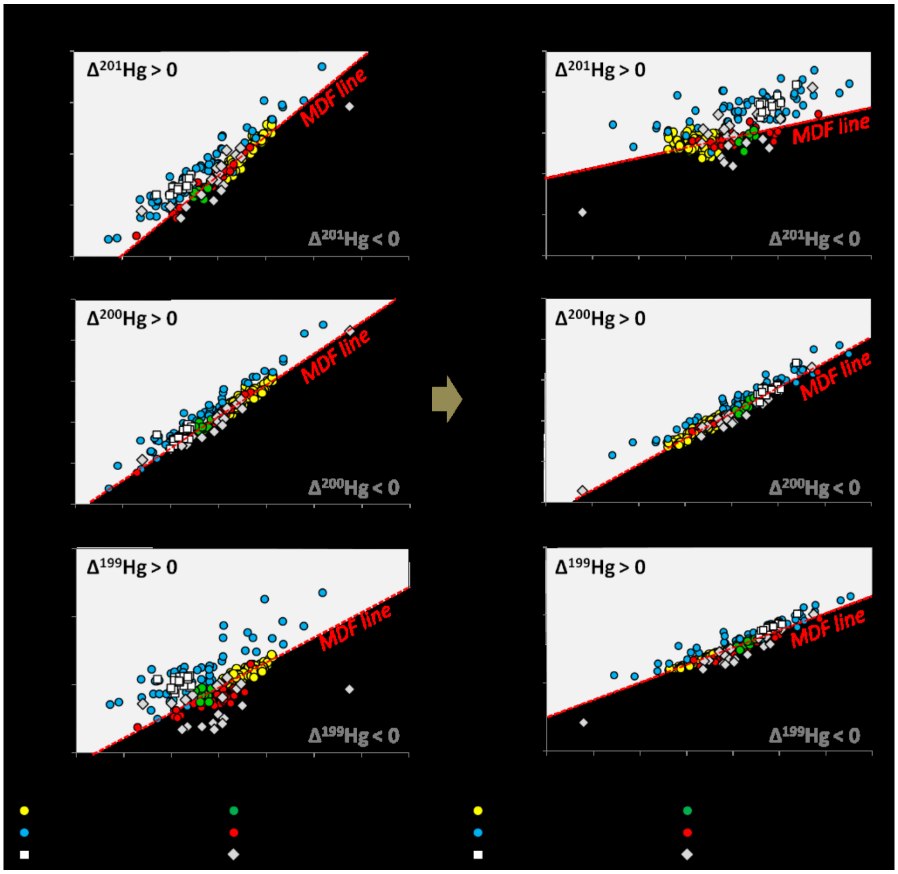

As shown on the right side of the figure, following the reviewer's suggestion, the Hg isotope ratios ($^{198}Hg/^{202}Hg$, $^{199}Hg/^{202}Hg$, $^{200}Hg/^{202}Hg$, $^{201}Hg/^{202}Hg$) remain linearly correlated with each other for NIST-3133 and UM-Almadén Hg standard solutions. More importantly, samples with positive MIF are also beyond the MDF lines, and samples with negative MIF are still underneath the MDF lines. Therefore, the renormalizing of the data does not unfortunately appear to provide a smoking gun.

If not, I suggest spending more time on the description of the HG data and how it is different and distinct and then to streamline the argument brought up later about its relationship to hypotheses that have been presented on the basis of the S and C data. I think a strong case can be made for the Hg being something important and new.

We thank the reviewer for this suggestion, and have restructured the manuscript in an attempt clarify the importance of the Hg-MIF data.

The data in S2 look like they could define a completely different field than the other data. It would be interesting to know if it was statistically resolved.

We thank the reviewer for pointing this out. We added a discussion of the potential importance of this difference in the manuscript (lines 201-207).

I think you undersell the significance of the Hg data and its use. The observations are striking and put another important constraint on the origin of the unusual S-C excursions you have documented previously.

We thank the reviewer for his support. As above, we have attempted to modify the text to focus on the importance of the Hg isotope data, and clarify this in the conclusions.

Reviewer #3 comments and responses:

Zerkle et al. present an interesting new dataset featuring both mercury and sulphur isotopes, and attempt to use these geochemical parameters to investigate atmospheric developments shortly prior to the GOE. I have not seen mercury isotopes used in this way before, so the idea is certainly novel and interesting, and has the insurance of using them alongside a much more established proxy of Archean atmospheric studies in the sulphur MIF.

We thank the reviewer for their support and have substantially modified the manuscript following their suggestions.

However, I feel that the interpretations presented by the authors are not always as strong as they suggest, and in some of the claims are unsubstantiated. Whilst they do acknowledge that both the mercury and sulphur cycles are complex and that there is much that we do not know about them (especially in the Archean, where both cycles were likely rather different), they rule out some of the possible controls on the mercury isotopes a little too easily. I don't think that they have as strong a case for ruling out volcanism and runoff as impactors as they claim.

As in response to R1 above, we have significantly modified the manuscript, including adding new and previously published data, to more thoroughly examine the potential for volcanism and runoff in controlling the Hg-MIF trends we see. In particular, we have modified our interpretation to allow for transient volcanism as a Hg source in high Hg and Hg/TOC sediments at the top of the mudstone interval and at the very bottom of the section. However, elemental weathering proxy data (new Figure S5) still argue against enhanced terrestrial runoff as a significant source of Hg to the section.

Also, there appears to be an inherent assumption made that because the perturbations in sulphur and mercury occur at the same level (829.22–838.78m depth), they must be directly related to the same process. This might be the case, but it doesn't have to be. I think that more thought about the non-atmospheric alternatives for the observed geochemical perturbations is needed, alongside additional data that can help rule out some of these other controls more conclusively, in order to strengthen the arguments presented here. Therefore, I cannot recommend that this manuscript be accepted by Nature Communications at this time.

We take the reviewer's point that these two proxies might not necessarily be responding to the same processes. As above, we have expanded our discussion to provide further consideration of alternative processes for producing the Hg-MIF trends, and allow for the contribution of volcanism as an important process in the upper part of the mudstone interval. However, we maintain that a variety of evidence (including S-MIF data) points towards changes in atmospheric chemistry as the dominant control on Hg-MIF values throughout the majority of this interval.

Major Comments:

The authors state that the change in Hg MIF is not entirely confined to the mudstone interval. Looking at figure 1, I am not at all convinced by this statement. At best, only the very bottom

and topmost samples occur just below and above, respectively, the mudstone, and they record the decline into and recovery from the excursions. Also can the authors be absolutely certain that there could not be some movement of mercury at the interface of the two different lithologies. Frustratingly, the two other mudstone intervals in the section have only one data point between the two of them, so it is impossible to judge what signal is recorded in the other mud units. If I'm wrong about this, then a zoomed-in stratigraphic figure showing just the main mudstone unit and a meter either side might help make this clear.

As in response to R1 above, we have added a more careful discussion of potential lithologic controls on the Hg isotope data. We have additionally rewritten the discussion to more thoroughly to examine all the possibilities that could have led to the negative shifts in Δ^{199}Hg and Δ^{200}Hg values across this interval (Lines 208-260).

The authors are not entirely correct to rule out the occurrence of volcanism on the absence of a Hg/TOC signal. Yes, many Phanerozoic igneous provinces have been linked to Hg/TOC peaks, but it has been shown that not all of them generate such a peak. Moreover, even for mega volcanic events that do cause Hg/TOC enrichments, the spikes do not always happen throughout the entire history of volcanism, and nor does every sedimentary record deposited at that time manifestly record them (see Percival et al., 2018, American Journal of Science; and Grasby et al., 2019, Earth Science Reviews). Of course, one could argue that if there isn't sufficient volcanic Hg arriving at this location to cause a Hg/TOC peak, how could there be enough to affect the Hg MIF? It's tricky, but one could envisage a scenario where TOC rises independently of Hg (note the change in lithology to mudstones which could allow this), in which case excess volcanic Hg devoid of MIF could arrive and change the sedimentary MIF composition, but the extra TOC cancel out any Hg/TOC peak. A slightly convoluted scenario, but not an impossible one.

We thank the reviewer for pointing this out. We agree with this scenario, and have revised our discussion and interpretation to allow for volcanic Hg as contributing to high Hg and Hg/TOC samples in the upper part of the mudstone interval (Lines 112-120).

However, I actually think that runoff of Hg is the more plausible alternative. The authors do mention runoff, but then rule it out on the basis that the terrestrial biosphere did not exist in the Precambrian to provide negative MIF from land. However, geogenic Hg is also thought to be devoid of MIF in both odd and even Hg isotopes (see Blum et al's 2014 review), and this could still have been present in terrestrial runoff in the Archean. A recent paper by Zheng et al. (2018, PNAS) also suggested that negative MIF of Hg could also arise through development of euxinic conditions, this should be mentioned as well.

As in our response to R1 above, we have additionally rewritten the discussion to more thoroughly to examine all the possibilities that could have led to the negative shifts Δ^{199}Hg and Δ^{200}Hg values across this interval, including terrestrial runoff (not supported by new elemental elemental proxy data; Figure S5) and development of euxinia (not supported by previously published Fe speciation data or total S contents; Figure 2A, S2).

Because a lithological change does seem to be a feature of most (if not all) of the stratigraphic level recording the Hg and S perturbations, such local perturbations should be considered in

more detail. The authors already mention Fe speciation as evidence for redox conditions during the core. I would suggest a new figure presenting this data, along with any further S data (S contents? d34S?) that can relate redox conditions and any connection between redox and Hg.

As requested, we have added previously published Fe speciation and pyrite $\delta^{34}S$ data for this interval to Figure S2, and cross plots of Hg-MIF versus total S contents in Figure 2A.

I would also strongly suggest presenting detrital proxy data (e.g., Ti/Al, Si/Al, Zr/Rb) in this figure to confirm what the role of terrestrial runoff (if any) might be.

We have also added detrital elemental proxy data, including K/Al, Si/Al, Ti/Al, and Zr/Al, as shown in Figure S5.

It is a pity that there is only one stratigraphic record presented here; a second record might have greatly strengthened the case against local environmental influences on these proxies. However, given the difficulties in even finding two demonstrably stratigraphically correlated Archean records, never mind the time-consuming nature of the analyses, incorporating a second site record is clearly beyond the scope of this work. But further analyses to highlight redox/runoff conditions would be very important.

We agree that additional records will be extremely useful in further testing the interpretation of changes in Hg-MIF across the mudstone interval. However, the occurrence of large-magnitude Hg-MIF throughout the majority of this section is independent of lithology and remains one of the most exciting aspects of this study, which has seemingly gotten drowned out by controversy over this one interval of change.

Finally, even if the authors are correct in their assessment that these perturbations are related to atmospheric changes, why do they have to be connected to an organic haze, and not a slight increase in atmospheric oxygen (leading to more ozone). This has been related to changes in S MIF previously, and 'whiffs' of oxygen have long been thought to occur in the latest Archean. Maybe that doesn't help explain the change in Hg MIF, but as noted above, there are other ways to bring in Hg devoid of MIF besides atmospheric fluctuations.

As discussed in the manuscript (Lines 256-260), ozone would completely shut down the formation of S-MIF, which remains at very high levels throughout this interval (as shown in Figure S2).

As above, the previous interpretation of the S-MIF data as representing a global atmospheric haze event relies on a significant body of previously published data from 5 cores on 2 continents, based on multi-proxy datasets that are conserved at the grain scale and independent of lithologies (Zerkle et al., 2012, Nature Geo; Farquhar et al., 2014, PNAS; Izon et al., 2015, EPSL; 2017, PNAS). We have further expanded our discussion of previous evidence for these events in the manuscript (Lines 261-285) as well as added a supplementary figure showing additional evidence published from this section and throughout the full GKF01 core (Figure S2). These additional sections will provide obvious targets for future Hg isotope work, but this is beyond the scope of the current study.

In summary, more detailed consideration of possible alternative controls on these perturbations, especially the Hg MIF, is needed before this manuscript can be accepted. Additional geochemical data highlighting the potential signals of changes in redox and terrestrial runoff should be presented as part of this. Because this represents significant changes to the manuscript, I cannot currently recommend it for publication in its current form.

As outlined in the responses above, we have significantly revised our discussion and interpretations and added an abundance of new and previously published data, which we hope will fully address the reviewer's concerns.

Minor comments:

L. 34: In this sentence, 'we' refers to 7 billion humans. Throughout the rest of the manuscript, 'we' refers to the authors. I suggest a rephrase in this context.

We changed this to "the planet", as requested.

L. 52: Some would define the atmosphere as part of the Earth's surface, in which case the transition of chemical species from the atmosphere to the Earth's surface doesn't work as a statement. I assume that the authors are referring to the aquatic and terrestrial realms when they talk about the Earth's surface. But this needs to be made clearer.

We disagree; the distinction seems obvious within the context of the sentence, and we would prefer to leave it simply as "Earth's surface" rather than listing all of the components.

L. 64: Change 'could' to 'would'. I think that one can fairly confidently state that Hg biogeochemical cycling would definitely have been rather different in the Precambrian.

The sentence has been changed accordingly.

L. 74: Include uptake by the terrestrial biosphere should be included here. Yes, I know that it is not applicable for Precambrian, but it should be stated for completeness. It can then be subsequently ruled out as a process of any importance in the context of this study.

"Direct uptake by vegetation and soils" is included in this paragraph.

L. 96–101: I suspect that the conclusion drawn here is correct, that the increased Hg concentration is related to increased burial efficiency by the additional organic carbon. But a note of caution should be issued, a number of the samples have rather low TOC contents. General protocol at the moment is to not use TOC values <0.2 wt% to normalize Hg against, as such low values carry comparatively large %uncertainties which consequently inflate the uncertainty in any Hg/TOC ratios. At the very least, the authors need to acknowledge this issue with some of the samples, and potentially remove the Hg/TOC ratios associated with those TOC values from the figure. As I say, in this case I don't think that it effects the author's conclusions, but it should be done to move the study in line with current practice.

We thank the reviewer for pointing this out. We have filtered the Hg/TOC data to only include samples with > 0.2 wt% TOC, as suggested.

L. 131–133: Somewhere here the study of Zheng et al. (2018, PNAS) about the control of photic zone euxinia on Hg odd isotope MIF needs to be mentioned.

As above, we have added this to our discussion of potential causes for the shift in Hg-MIF values.

L. 159: The exact mechanism of depositing Hg(0) should be clarified. Direct take up by vegetation is not an option in the Precambrian, and depositing a gas (the chief form of Hg0 in the atmosphere) is rather counterintuitive concept.

We have modified the discussion to clarify that gaseous Hg(0) with negative $\Delta^{199}Hg$ and $\Delta^{200}Hg$ was oxidized by organic compounds in the atmosphere and then deposited to the ocean (Lines 254-256).

L. 180–182: As I mentioned above, I am not completely convinced by this statement that the Hg MIF extends to beyond the mudstone unit. At best, only the topmost and bottommost samples do, and there are background samples that register no/negative MIF anyway. Also the other lithologies do feature mud-rich strata as well according to the key in figure 1. I suggest that a new figure focusing on the mudstone interval and a few meters either side (possibly with a more detailed lithological column) would help address this.

As in response to R1 above, we have modified this statement and the discussion accordingly.

L. 183–186: Contribution of Hg from runoff of terrestrial vegetation and organic matter would indeed have presumably been negligible during the Precambrian. But what about geogenic Hg in runoff. This is also (albeit on the basis of a limited number of studies) devoid of MIF, so an increase in terrestrial runoff might also shift the sedimentary composition of MIF towards 0, and also potentially increase the clay influx to this marine setting that would be reflected by the observed change in lithology.

As above, we have expanded our discussion of terrestrial runoff as a potential cause for the shift in Hg-MIF (Lines 225-235).

L. 207: Volcanic emissions with no Hg MIF are based on studies of a single volcanic system, which is an arc system. There is no guarantee that this is representative of volcanism as a whole today, let alone in the Archean when plate tectonics was rather different. Of course, these are the data that we have, so we have to make that assumption for now, but at the very least, the authors should be clear about the limitations of this assumption.

We disagree with the statement that plate tectonics was necessarily “rather different” in the Archean. The timing of the initiation of modern-style plate tectonics is highly controversial, with estimates spanning over 1 Ga (e.g., see 2018 Philosophical Transactions of the Royal Society A, Volume 376, Issue 2132: papers from discussion meeting ‘Earth dynamics and the development

of plate tectonics'). A detailed discussion of these arguments is beyond the scope of this paper; however, the majority of estimates suggest this was occurring as early as 3.0 Ga, well before the deposition of these sediments. Nevertheless, we have added a disclaimer to the discussion, stating "assuming that Archean volcanic Hg emmissions had similar isotope values to today" (Lines 248-249).

L. 207–208: This is not entirely correct. Some Phanerozoic Large Igneous Provinces have been claimed as recorded by Hg/TOC peaks, but this is not the case for all such volcanic events, and even when Hg/TOC peaks are observed, most case studies show that not all sedimentary records coeval with those eruptions will show the enrichments. So whilst one should certainly not infer a volcanic influence just on the basis of this dataset, neither can one rule it out because there is not a Hg/TOC spike.

As above, we have expanded our consideration of volcanism as a contributor to changes in Hg-MIF values and further examine the associated Hg/TOC data.

L. 210–214: Redox conditions are discussed here. It would be good to put these into a figure (along with runoff proxies such as Ti/Al, Si/Al, Zr/Rb) so that stratigraphic trends can be compared to the Hg- and S-isotope data. This can then be used to clarify whether or not there is any relationship between these changes in MIF and local environmental conditions.

As above, we have added previously published Fe speciation and $\delta^{34}S$ data (Figure S2), and new data for total S content (Figure 2A), and terrestrial runoff proxies (Figure S5).

REVIEWERS' COMMENTS:

Reviewer #1 (Remarks to the Author):

I saw the value in these data and this manuscript immediately, although I did have some misgivings about the strength/uniqueness of some of the conclusions. Two things have allayed those concerns. First, I agree that this is an exceptional data set that should be brought to light. These are novel/unprecedented results that speak to important issues. Second, the authors have done a remarkable job of addressing my comments and the many excellent suggestions from the other two reviewers. There is balance and rigor throughout. The revision is comprehensive, which was no easy task given space limitations. I am convinced a stronger paper has resulted. I support moving toward publication.

Reviewer #2 (Remarks to the Author):

I have read the revised manuscript, reviews, and response.
The authors describe an observation that an increase in Hg MIF is observed for both odd and even Hg isotopes in late Archean age sediments from the GKF01 core when compared to Hg-MIF reported for post-Archean sediments (Their Figure 3 and other figures). The authors consider various sources of Hg MIF learned from prior studies and note that even isotope Hg MIF is only known for atmospheric reactions. The manuscript interprets this observation to be of an atmospheric origin on the basis of Hg MIF involving even isotopes, which are not known to be produced by non atmospheric processes. Given that the Hg MIF involving odd isotopes is correlated with the Hg MIF involving even isotopes, the authors assert that both odd and even isotope Hg MIF have an atmospheric origin. The authors then argue that different magnitude of Hg MIF for the Late Archean succession relative to that observed for post Archean successions reflects differences in the atmospheric chemistry of the late Archean compared to more recent geologic time. My impression is that the data are of high quality and reveal a real difference in Hg MIF between the successions and that the interpretation of a change in Hg-MIF is supported by the data and that the attribution of this Hg MIF to an atmospheric origin that was somehow different than seen in the post Archean is not only plausible, but likely the most straightforward explanation. The late Archean atmosphere is thought to have been nearly devoid of oxygen and to have had a very different chemistry and to have transitioned to a more modern-like atmosphere in several steps that started shortly after the end of the Archean.
Building on this first-order observation and interpretation, the manuscript discusses relationships that appear to exist between Hg MIF and other geochemical characteristics of the studied GKF01 sedimentary succession. They note a change in the Hg MIF that is associated with a change in S MIF that has previously been documented by previous studies and attributed to a change in late Archean atmosphere chemistry associated with the formation of an organic Haze. They suggest these changes have a common origin and which would imply that the Hg MIF thus carries additional information about the late Archean atmosphere. They also examine relationships between Hg content and total organic carbon (TOC) and sedimentary facies to evaluate the possibility that the signals could reflect other causes related to volcanism or facies or runoff and make the case that the most parsimonious explanation involves a change in the Atmosphere. I find their arguments measured and convincing.

Reviewer #3 (Remarks to the Author):

The authors have done a good job in addressing many of my concerns relating to the original submission of the manuscript. There are some comments/issues that I have raised in this review of the revised manuscript, but these are fairly minor points. If they can be addressed to the editor's satisfaction, then I would be happy for this revised version of the manuscript to be accepted.

My one remaining major point concerns the potential role (or not) of volcanism in the production of the Hg-MIF. In my earlier review I indicated that the reviewers might be ruling out the role of volcanism a little too swiftly. However, if anything they have now gone too far the other way, and seem to be too confident in the 3 high Hg content samples (which also form a Hg/TOC peak) potentially being volcanism, and that the rest of the 830–840m window is probably not volcanic because of the low Hg/TOC values. My point was that on its own, the Hg/TOC ratio is not axiomatically reliable as a proxy for volcanism unless there is other evidence for volcanic activity at approximately the same time. I don't think that the authors need to change their ultimate conclusion (that volcanism isn't the main driver of the MIF). But maybe to restructure/rephrase the argument between L. 236–249. Start with there being low Hg and Hg/TOC values for much of the 840–830m strata, which suggests that if there was any volcanism, it likely did not greatly perturbed the Hg cycle. Then go on to say that the small Hg/TOC peak might be consistent with an influx of volcanic Hg, but that there is no supporting evidence for volcanism from the REE patterns, and in any case, the lack of correlation between the Hg/TOC peak and drop in MIF values means that even if the Hg/TOC peak is volcanically derived, the volcanism can't be solely responsible for the Hg-cycle perturbation. This might be what the authors are trying to say at the moment, but at the moment it's not coming across for me.

Minor comments:

Lines 35–37: 'significant changes' would be better than 'a significant transition' for me (especially since the abstract has the similar phrase 'a dramatic transition' in its first sentence). Also the word 'atmosphere' appears three times in this first sentence of the introduction, which is a little clumsy, so a bit of rephrasing would be good.

L. 48: 'large $\Delta^{33}S$ and $\Delta^{36}S$ values'. 'Large values' is a strange wording for me; do the authors mean 'high values' or 'large excursions' or both? Correct as appropriate.

L. 42–58: There is a lot of detail here on how the atmosphere (and especially a reduced atmosphere) can affect S-isotope compositions. But I believe that marine processes and redox changes can also have an effect. Briefly mentioning these non-atmospheric controls in a couple of sentences would be useful.

L. 64: Change 'long atmospheric residence time (0.5 to 2 years), allowing for global transport' to 'sufficiently long atmospheric residence time (0.5 to 2 years) for global transport'. Because in absolute terms, 0.5–2 years isn't very long, the point is that it's long enough for Hg to be widely dispersed through the stratosphere (at least in the modern).

L. 69: Add 'typically' to '…ultimate burial is to ocean sediments…', as Hg can sometimes end up in terrestrial sediments such as at the bottom of lakes (for example).

L. 113–119: This is a little bit oversimplified for me in terms of what the Hg/TOC may mean. First off, I would change the first sentence to read 'Since volcanic emissions commonly (though not always) shift Hg/TOC ratios in sediments, these data might indicate that…' in order to clarify that not too much reliance is being placed on the ratio. Secondly, I would change 'consistent with volcanic Hg emissions' to 'consistent with a major influx of Hg from an external source, such as increased volcanic activity.' I don't think that the following sentence of 'These three anomalously

high Hg samples could point to an additional, transient volcanic input of Hg.' is required, as it just repeats the point made in the previous sentence.

L. 148: The Blum et al. (2014) review of Hg isotopes would be the better article to cite here than the Thibodeau and Bergquist overview.

L. 158–159: Clarify that these atmospheric even-number MIF observations are modern-day studies rather than paleoatmosphere studies.

L. 172–174: The correlation could indeed indicate that both the even- and odd-number MIF is mainly produced in the atmosphere. But is this the only explanation? Could there not be a scenario whereby both atmospheric and sea-surface photoreduction reactions were increased simultaneously (for instance if there was a change in the atmospheric composition that might have allowed different wavelengths of light to reach the Earth's surface)? In this case, whilst the even-number MIF would still have been occurring in the atmosphere, the odd-number MIF would have been generated in both the atmospheric and marine realms. My point is that just because even-number MIF is (we think) exclusively generated in the atmosphere, that doesn't manifestly mean that both of the correlative odd- and even-number MIFs were generated atmospherically, as odd-number MIF could have been produced in either the atmosphere or surface ocean.

L. 204–205: Again, the conclusion of Hg-MIF production being different in anoxic versus oxygenated atmospheres may well be correct, but this is not the only interpretation. Since the whole MIF-MDF field is different, presumably it could also point to differences in MDF generation in a Precambrian world? This should also be mentioned for the sake of completeness.

L. 218: Change to 'enhanced terrestrial input of geogenic Hg' to re-emphasize the point that this is not Hg derived from plants and soils (as it would be in the modern world).

L. 221: Change to 'As noted above'

L. 225–228: Careful. Organic-rich mudstones can develop in conditions where increased terrestrial runoff is occurring, particularly if that runoff is bringing in nutrients that promote increased primary productivity and therefore organic carbon burial (especially in an Archean ocean where organic-matter preservation would have been much greater). So the lithology change, in and of itself, does not axiomatically mean that terrestrial runoff could not have been elevated, although nor does it mean it definitely was. Also, given that the text refers to basinal deepening, do the authors mean 'a more proximal depositional environment' on line 226? Or 'a more distal depositional environment'?

L. 233–235: This other mudstone unit up-core has only one data point within it, I think (two at the most). This is a shortcoming which should be acknowledged in this sentence.

L. 262–264: The carbon-isotope data aren't on Figure 1, but they are on Figure S2. So Figure S2 should also be referred to here.

L. 269: What is meant by 'grain-scale' in this context?

L. 309–310: 'We have...' would sound better than 'We've...' (the latter sounds rather casual). Also I think that rather more care is needed when promoting Hg-MIF as a paleoatmospheric proxy in marine sediments. At best, this could only ever truly apply to even-number Hg MIF, assuming that our current observations of it only taking place in the atmosphere are representative, as we already know that odd-number MIF can be produced in aquatic settings. Also, does this proxy only apply in the Precambrian? Presumably once a terrestrial biosphere arises that introduces an extra source of MIF? Or is it even limited just to the Archean before atmospheric oxygen levels arise? Essentially, I think that whilst this paper shows that Hg-MIF can be a useful indication of

atmospheric changes on the Early Earth, to say it is a powerful paleoatmospheric proxy is rather misleading as it implies a much more general applicability than I suspect is the case. So this wording needs to be toned down.

Figure 4: There is no explanation of what the different colors of text (e.g. red Volcanic Hg(0)) mean in the figure? I assume there is some significance, so please make clear either in the figure or in the caption what the colors mean. Also, I personally think that it would be nice to illustrate the proposed haze on Figure 4B, in order to make its influence in that scenario clear.

RESPONSE TO REVIEWERS' COMMENTS:

Reviewer #1 (Remarks to the Author):

I saw the value in these data and this manuscript immediately, although I did have some misgivings about the strength/uniqueness of some of the conclusions. Two things have allayed those concerns. First, I agree that this is an exceptional data set that should be brought to light. These are novel/unprecedented results that speak to important issues. Second, the authors have done a remarkable job of addressing my comments and the many excellent suggestions from the other two reviewers. There is balance and rigor throughout. The revision is comprehensive, which was no easy task given space limitations. I am convinced a stronger paper has resulted. I support moving toward publication.

We thank the reviewer for their support.

Reviewer #2 (Remarks to the Author):

I have read the revised manuscript, reviews, and response.
The authors describe an observation that an increase in Hg MIF is observed for both odd and even Hg isotopes in late Archean age sediments from the GKF01 core when compared to Hg-MIF reported for post-Archean sediments (Their Figure 3 and other figures). The authors consider various sources of Hg MIF learned from prior studies and note that even isotope Hg MIF is only known for atmospheric reactions. The manuscript interprets this observation to be of an atmospheric origin on the basis of Hg MIF involving even isotopes, which are not known to be produced by non atmospheric processes. Given that the Hg MIF involving odd isotopes is correlated with the Hg MIF involving even isotopes, the authors assert that both odd and even isotope Hg MIF have an atmospheric origin. The authors then argue that different magnitude of Hg MIF for the Late Archean succession relative to that observed for post Archean successions reflects differences in the atmospheric chemistry of the late Archean compared to more recent geologic time. My impression is that the data are of high quality and reveal a real difference in Hg MIF between the successions and that the interpretation of a change in Hg-MIF is supported by the data and that the attribution of this Hg MIF to an atmospheric origin that was somehow different than seen in the post Archean is not only plausible, but likely the most straightforward explanation. The late Archean atmosphere is thought to have been nearly devoid of oxygen and to have had a very different chemistry and to have transitioned to a more modern-like atmosphere in several steps that started shortly after the end of the Archean.

Building on this first-order observation and interpretation, the manuscript discusses relationships that appear to exist between Hg MIF and other geochemical characteristics of the studied GKF01 sedimentary succession. They note a change in the Hg MIF that is associated with a change in S MIF that has previously been documented by previous studies and attributed to a change in late Archean atmosphere chemistry associated with the formation of an organic Haze. They suggest these changes have a common origin and which would imply that the Hg MIF thus carries additional information about the late Archean atmosphere. They also examine relationships between Hg content and total organic carbon (TOC) and sedimentary facies to evaluate the possibility that the signals could reflect other causes related to volcanism or facies or runoff and make the case that the most parsimonious explanation involves a change in the Atmosphere. I find their arguments measured and convincing.

We thank the reviewer for his support.

Reviewer #3 (Remarks to the Author):

The authors have done a good job in addressing many of my concerns relating to the original

submission of the manuscript. There are some comments/issues that I have raised in this review of the revised manuscript, but these are fairly minor points. If they can be addressed to the editor's satisfaction, then I would be happy for this revised version of the manuscript to be accepted.

We thank the reviewer for their support.

My one remaining major point concerns the potential role (or not) of volcanism in the production of the Hg-MIF. In my earlier review I indicated that the reviewers might be ruling out the role of volcanism a little too swiftly. However, if anything they have now gone too far the other way, and seem to be too confident in the 3 high Hg content samples (which also form a Hg/TOC peak) potentially being volcanism, and that the rest of the 830–840m window is probably not volcanic because of the low Hg/TOC values. My point was that on its own, the Hg/TOC ratio is not axiomatically reliable as a proxy for volcanism unless there is other evidence for volcanic activity at approximately the same time. I don't think that the authors need to change their ultimate conclusion (that volcanism isn't the main driver of the MIF).

But maybe to restructure/rephrase the argument between L. 236–249. Start with there being low Hg and Hg/TOC values for much of the 840–830m strata, which suggests that if there was any volcanism, it likely did not greatly perturbed the Hg cycle. Then go on to say that the small Hg/TOC peak might be consistent with an influx of volcanic Hg, but that there is no supporting evidence for volcanism from the REE patterns, and in any case, the lack of correlation between the Hg/TOC peak and drop in MIF values means that even if the Hg/TOC peak is volcanically derived, the volcanism can't be solely responsible for the Hg-cycle perturbation. This might be what the authors are trying to say at the moment, but at the moment it's not coming across for me.

We thank the reviewer for this suggestion - the discussion of volcanic inputs to the Hg-MIF values has been restructured, as follows:

"An increase in volcanic Hg input across this interval (scenario 3) cannot explain the change in Hg-MIF values either. Hg concentrations and Hg/TOC remain generally low in these strata, suggesting that even if there were volcanism it did not greatly perturb the Hg cycle. Discrete peaks in Hg levels and Hg/TOC could indicate periodically enhanced volcanic Hg emissions22, but REE patterns further suggest that volcanism did not typify this sedimentary interval24. Regardless, the lack of correlation between the peak in Hg/TOC and the change in Hg-MIF means that even if the Hg/TOC peak was volcanically derived, the volcanism was not solely responsible for the Hg cycle perturbation."

Minor comments:

Lines 35–37: 'significant changes' would be better than 'a significant transition' for me (especially since the abstract has the similar phrase 'a dramatic transition' in its first sentence). Also the word 'atmosphere' appears three times in this first sentence of the introduction, which is a little clumsy, so a bit of rephrasing would be good.

The sentence has been modified, as follows: Earth's atmosphere has undergone significant changes during the 4.5 billion-year history of the planet, from fully anoxic and reducing in the Archean, to the oxygen-rich atmosphere of today.

L. 48: 'large Δ33S and Δ36S values'. 'Large values' is a strange wording for me; do the authors mean 'high values' or 'large excursions' or both? Correct as appropriate.

Neither of the suggested changes is technically correct. We have modified this to "large magnitude", since these include both large positive and large negative values, consistently preserved throughout the Archean (not as excursions).

L. 42–58: There is a lot of detail here on how the atmosphere (and especially a reduced atmosphere) can affect S-isotope compositions. But I believe that marine processes and redox changes can also have an effect. Briefly mentioning these non-atmospheric controls in a couple of sentences would be useful.

S-MIF is only produced via atmospheric processes – marine biogeochemical cycling and redox changes have negligible effects on these values.

L. 64: Change ‘long atmospheric residence time (0.5 to 2 years), allowing for global transport’ to ‘sufficiently long atmospheric residence time (0.5 to 2 years) for global transport’. Because in absolute terms, 0.5–2 years isn’t very long, the point is that it’s long enough for Hg to be widely dispersed through the stratosphere (at least in the modern).

The sentence has been modified as requested.

L. 69: Add ‘typically’ to ‘…ultimate burial is to ocean sediments…’, as Hg can sometimes end up in terrestrial sediments such as at the bottom of lakes (for example).

The sentence has been modified as requested.

L. 113–119: This is a little bit oversimplified for me in terms of what the Hg/TOC may mean. First off, I would change the first sentence to read ‘Since volcanic emissions commonly (though not always) shift Hg/TOC ratios in sediments, these data might indicate that…’ in order to clarify that not too much reliance is being placed on the ratio. Secondly, I would change ‘consistent with volcanic Hg emissions’ to ‘consistent with a major influx of Hg from an external source, such as increased volcanic activity.’ I don’t think that the following sentence of ‘These three anomalously high Hg samples could point to an additional, transient volcanic input of Hg.’ is required, as it just repeats the point made in the previous sentence.

The discussion has been modified as requested.

L. 148: The Blum et al. (2014) review of Hg isotopes would be the better article to cite here than the Thibodeau and Bergquist overview.

The reference has been modified as requested.

L. 158–159: Clarify that these atmospheric even-number MIF observations are modern-day studies rather than paleoatmosphere studies.

The sentence has been modified as requested.

L. 172–174: The correlation could indeed indicate that both the even- and odd-number MIF is mainly produced in the atmosphere. But is this the only explanation? Could there not be a scenario whereby both atmospheric and sea-surface photoreduction reactions were increased simultaneously (for instance if there was a change in the atmospheric composition that might have allowed different wavelengths of light to reach the Earth’s surface)? In this case, whilst the even-number MIF would still have been occurring in the atmosphere, the odd-number MIF would have been generated in both the atmospheric and marine realms. My point is that just because even-number MIF is (we think) exclusively generated in the atmosphere, that doesn’t manifestly mean that both of the correlative odd- and even-number MIFs were generated atmospherically, as odd-number MIF could have been produced in either the atmosphere or surface ocean.

We prefer to present the most likely scenario, rather than to speculate on unknown mechanisms (and the scenario presented by the reviewer still requires a change in atmospheric chemistry to be viable). We believe the words "implies" and "mainly" in the sentence sufficiently allow that there are potentially other options.

L. 204–205: Again, the conclusion of Hg-MIF production being different in anoxic versus oxygenated atmospheres may well be correct, but this is not the only interpretation. Since the whole MIF-MDF field is different, presumably it could also point to differences in MDF generation in a Precambrian world? This should also be mentioned for the sake of completeness.

This statement is specifically about Hg-MIF, which is only produced in the atmosphere, therefore it is correct to state that changes in MDF in the Precambrian would not affect this signal.

L. 218: Change to 'enhanced terrestrial input of geogenic Hg' to re-emphasize the point that this is not Hg derived from plants and soils (as it would be in the modern world).

The sentence has been modified as requested.

L. 221: Change to 'As noted above'

The sentence has been modified as requested.

L. 225–228: Careful. Organic-rich mudstones can develop in conditions where increased terrestrial runoff is occurring, particularly if that runoff is bringing in nutrients that promote increased primary productivity and therefore organic carbon burial (especially in an Archean ocean where organic-matter preservation would have been much greater). So the lithology change, in and of itself, does not axiomatically mean that terrestrial runoff could not have been elevated, although nor does it mean it definitely was. Also, given that the text refers to basinal deepening, do the authors mean 'a more proximal depositional environment' on line 226? Or 'a more distal depositional environment'?

Organic-rich mudstones in these cores were interpreted by Schroder et al. (2006) as forming in a deeper water depositional environment (as referenced in the statement). We thank the reviewer for pointing out our mistaken terminology; this has been modified in the text.

L. 233–235: This other mudstone unit up-core has only one data point within it, I think (two at the most). This is a shortcoming which should be acknowledged in this sentence.

The discussion has been modified as requested.

L. 262–264: The carbon-isotope data aren't on Figure 1, but they are on Figure S2. So Figure S2 should also be referred to here.

Figure S2 is already referred to here.

L. 269: What is meant by 'grain-scale' in this context?

Within context, the sentence states that the signal is "conserved at the grain-scale", meaning the same signal is seen at the spatial scale of individual grains.

L. 309–310: 'We have…' would sound better than 'We've…' (the latter sounds rather casual). Also I think that rather more care is needed when promoting Hg-MIF as a paleoatmospheric proxy in marine sediments. At best, this could only ever truly apply to even-number Hg MIF, assuming that our current observations of it only taking place in the atmosphere are representative, as we already know that odd-number MIF can be produced in aquatic settings. Also, does this proxy only apply in the

Precambrian? Presumably once a terrestrial biosphere arises that introduces an extra source of MIF? Or is it even limited just to the Archean before atmospheric oxygen levels arise? Essentially, I think that whilst this paper shows that Hg-MIF can be a useful indication of atmospheric changes on the Early Earth, to say it is a powerful paleoatmospheric proxy is rather misleading as it implies a much more general applicability than I suspect is the case. So this wording needs to be toned down.

The sentence has been modified as requested. However, we disagree with the reviewer's suggestion that this is not a powerful paleoatmospheric proxy, given that we have shown it can provide important, independent evidence for changes in atmospheric chemistry (and it is only the second such proxy that can do so!).

Figure 4: There is no explanation of what the different colors of text (e.g. red Volcanic Hg(0)) mean in the figure? I assume there is some significance, so please make clear either in the figure or in the caption what the colors mean. Also, I personally think that it would be nice to illustrate the proposed haze on Figure 4B, in order to make its influence in that scenario clear.

The figure caption has been modified, as requested.
